# Carbon Ion Radiation Inhibits Glioma and Endothelial Cell Migration Induced by Secreted VEGF

**DOI:** 10.1371/journal.pone.0098448

**Published:** 2014-06-03

**Authors:** Yang Liu, Yuanyuan Liu, Chao Sun, Lu Gan, Luwei Zhang, Aihong Mao, Yuting Du, Rong Zhou, Hong Zhang

**Affiliations:** 1 Department of Radiation Medicine, Institute of Modern physics, Chinese Academy of Sciences, Lanzhou, China; 2 Key Laboratory of Heavy Ion Radiation Biology and Medicine of Chinese Academy of Sciences, Lanzhou, China; 3 Key Laboratory of Heavy Ion Radiation Medicine of Gansu Province, Lanzhou, China; 4 Graduate School of Chinese Academy of Sciences, Beijing, China; 5 Lanzhou University, Hospital of Stomatology, Lanzhou, China; Wayne State University School of Medicine, United States of America

## Abstract

This study evaluated the effects of carbon ion and X-ray radiation and the tumor microenvironment on the migration of glioma and endothelial cells, a key process in tumorigenesis and angiogenesis during cancer progression. C6 glioma and human microvascular endothelial cells were treated with conditioned medium from cultures of glioma cells irradiated at a range of doses and the migration of both cell types, tube formation by endothelial cells, as well as the expression and secretion of migration-related proteins were evaluated. Exposure to X-ray radiation-conditioned medium induced dose-dependent increases in cell migration and tube formation, which were accompanied by an upregulation of *vascular endothelial growth factor* (*VEGF*) and *matrix metalloproteinase* (*MMP*)*-2* and *-9* expression. However, glioma cells treated with conditioned medium of cells irradiated at a carbon ion dose of 4.0 Gy showed a marked decrease in migratory potential and *VEGF* secretion relative to non-irradiated cells. The application of recombinant *VEGF165* stimulated migration in glioma and endothelial cells, which was associated with increased FAK phosphorylation at Tyr861, suggesting that the suppression of cell migration by carbon ion radiation could be via *VEGF*-activated FAK signaling. Taken together, these findings indicate that carbon ion may be superior to X-ray radiation for inhibiting tumorigenesis and angiogenesis through modulation of *VEGF* level in the glioma microenvironment.

## Introduction

Malignant gliomas are the most common and lethal type of primary glioma in adults [1] due to its aggressivity and high propensity for invasion into surrounding normal tissue, which contribute to poor prognosis. Radiotherapy, a standard adjuvant to surgery, improves survival rates in patients, but resistance to treatment by some gliomas limits the success of clinical application [2].

High linear energy transfer (LET) heavy ions such as carbon ions have attracted attention as alternatives to conventional radiation for radiotherapy—particularly for tumors that are radiation-resistant under hypoxic conditions—owing to their superior physical characteristics and high lethality for tumor cells compared to low-LET radiation such as X- and γ-rays [3]–[4]. Our own studies have shown that carbon ion radiotherapy offers a high degree of control for targeting tumors and increases progression-free survival rates without significant radiation-induced toxicity in skin carcinoma patients [5].

Emerging evidence indicates that the tumor microenvironment contributes to radiation resistance [6] by regulating the levels of cytokines and growth factors, including *vascular endothelial growth factor* (*VEGF*) [7], *epidermal growth factor* (*EGF*) [8], *hepatocyte growth factor* (*HGF*) [9], and *basic fibroblast growth factor* (*bFGF*) [10], and promoting extracellular matrix degradation by *matrix metalloproteinases* (*MMPs*) [11].

Due to the scarcity of particle accelerators that can produce heavy ion radiation, it has not yet been established whether carbon ions can modify the glioma microenvironment and affect the migratory behavior of cells during tumor progression and metastasis. To address this question, the present study evaluated the contribution of carbon ion radiation to the motility of glioma and endothelial cells using tumor-conditioned culture medium as an inducer.

## Materials and Methods

### Cell culture techniques

C6 glioma cells (American Type Culture Collection) were grown in Ham's F-12 media (Gibco, Invitrogen Crop, USA) supplemented with 10% fetal bovine serum (FBS, HyClone, China). Human microvascular endothelial cells (HMEC-1, American Type Culture Collection) were grown in a modified MCDB 131 (Sigma Chemical, St. Louis, MO, USA) medium containing 20% FBS and 1.0 µg/ml hydrocortisone (Sigma), 10 ng/ml human epidermal growth factor (EGF, Sigma). All cells were cultured in 5% CO_2_ in humidified air at 37°C.

### Irradiation Procedure

C6 glioma cells were irradiated by heavy ion beams and X-rays. Carbon ion irradiation was performed by the equipment at the Heavy Ion Research Facility in Lanzhou (HIRFL-CSR, Institute of Modern Physics, Chinese Academy of Sciences, Lanzhou, China). Glioma cells were exposed to carbon ion irradiation at energy 350 MeV u^−1^ and LET 15.4 keV µm^−1^, with a dose rate of 0.5 Gy min^−1^. X-rays were from a cabinet X-ray irradiation system (100 kpv, 3 mA, beryllium window thickness 0.76 mm, Faxitron X-ray corp.). The dose rate was set to about 1.3 Gy/min. The film-to-source distance was set to 40.6 cm. Glioblastoma cells were irradiated with 0, 2.0, 4.0, 8.0 Gy.

### Tumor cell conditioned medium (TCM) and Irradiated conditioned medium (ICM) preparation

To generate TCM, C6 glioma cells were seeded at a density of 5×10^5^ cells/25 cm^2^ culture flasks. At 90% confluence of cell monolayer, culture medium was discarded to remove growth factors. Thereafter, cells were rinsed with saline solution and fed with fresh serum-free medium. TCM were collected from sham-irradiated cells considered as control conditioned medium. To obtain the ICM, cells were irradiated with carbon ion beams or X-rays under sterile conditions. And then irradiated cells were immediately rinsed three times with saline solution and fed with fresh serum-free medium. After 24 h of incubation, the supernatants was harvested and centrifuged at 2000×g for 15 min and passed through a 0.22 µm filter to remove cells and cell debris. This supernatant was stocked in 1.5 ml tubes and kept at −80°C.

### Transwell migration assays

The *in vitro* cell migration assays were performed by using a modified Boyden chamber inserted with polyethylene terephthalate filter membrane containing 8 µm pores in 24-well plates (Millipore, Milan, Italy). The upper chamber contained C6 glioma cells or HMEC-1 cells in serum-free culture medium, and the lower chamber contained TCM or ICM. After 24 h of incubation, migrated cells were fixed in 100% methanol for 1 h and then stained with Giemsa stain (Sigma) for 10 min and images were processed using AxioVs40 software (Ver. 4.8.0.0, Carl Zeiss, Germany). Data were obtained by measuring ten randomly selected fields of transwell-migrated cells using Image-Pro Plus 6.0 software (Media Cybernetics, Silver Spring, MD, USA).

### Gene expression analysis

Total RNA was extracted form glioma cells at 24 h after carbon ion or X-ray irradiation using the TRIzol Reagent (Invitrogen Life Technologies, Carlsbad, CA, USA) according to the manufacturer's instructions. The cDNA was synthesized from 1 µg total RNA using RT reagent kit with gDNA Eraser (Takara, Tokyo, Japan) following the manufacturer's protocols. Real-time polymerase chain reaction (quantitative PCR) was carried out the SYBR Premix EX Taq II kit (Takara, China) on an FTC-3000+instrument (Funglyn Biotech INC, Toronto, ON, Canada). Gene expression was detected using qPCR primers for VEGF and β-actin which was used to normalize the mRNA and cDNA quantity and quality. Sequences of the primers are as follows: VEGF sense, 5′-GATCATGCGGATCAAACCTCACC-3′, and antisense, 5′-CCTCCGGACCCAAAGTGCTC-3′; β-actin sense, 5′-TGAGCGCAAGTACTCTGTGTGGAT-3′, and antisense, 5′-TAGAAGCATTTGCGGTGCACGATG-3′. The PCR program was denatured at 95°C for 30 s, followed by 40 cycles of 95°C for 5 s, 60°C for 30 s. The fold changes of gene expression of the treatment groups were calculated by the 2^-ΔΔCt^ method [12].

### Lactate dehydrogenase (LDH) assay

The LDH leakage was determined by using commercially available kits (Beyotime Institute of Biotechnology, China). The cells were washed twice with PBS and then lysed to release the intracellular LDH into the new supernatant at 24 h after exposure to radiation. The supernatant (120 µl) was mixed with 60 µl of LDH reagent solution, sheltered from light and incubated at room temperature for 30 min. The LDH activity of the medium (LDH medium) and the cell lysate (LDH cells) were determined using a colorimetric assay at an absorbance wavelength of 490 nm and a reference wavelength of 630 nm using an Infinite M200 microplate reader (TECAN, Switzerland). The measured LDH activities were calculated by percentage of LDH released in the supernatant to that of cell lysates from intact cells (% LDH released).

### Wound-healing assay

Cells were seeded in a six-well plate at a concentration of 5×10^5^ per well and allowed to form a confluent monolayer. The layer of cells was scratched with a 1000 µl micropipette tip. Cells were then washed twice with fresh medium and replaced with TCM or ICM. Cells migrated into the wounded area, and photographs were taken immediately (0 h) and 24 h. The ability of cell motility was calculated by Image-Pro Plus 6.0 software (Media Cybernetics, Silver Spring, MD, USA) in the following way: the area covered by the migrating cells (24 h)/the wound area (0 h).

### Tube formation assay

96-well plates were coated with 60 µl of Matrigel (BD Biosciences, San Jose, CA) and allowed to solidify at 37°C for 1 h. After the Matrigel solidified, HMEC-1 endothelial cells (8×10^5^ cells/ml) were added in 200 µl of TCM or ICM. The cells were incubated at 37°C with humidified 95% air/ 5% CO_2_ for 8 h and photographed with the Carl Zeiss. Five randomly selected microscopic tube length was assayed by Image-Pro Plus 6.0 software (Media Cybernetics, Silver Spring, MD, USA).

### Cytokine protein quantification

We measured tumor-derived secreted cytokines according to the manufacturer's instructions. *VEGF* (ExCell Biology, Inc., Shanghai, China), *MMP*-*2* and *MMP*-*9* (Sangon Biotech, Shanghai, China) in the TCM or ICM of C6 glioma cells were detected by enzyme-linked immunosorbent assay (ELISA).

### Western blot analysis

Protein sample preparation was basically performed as previously described [13]. Protein samples were load onto 10% acrylamide gels for Sodium dodecyl sulfate-polyacrylamide gel electrophoresis (SDS-PAGE), and then the proteins were transferred to polyvinylidine difluoride membranes (Millipore Corporation, USA). The membrane was blocked and subsequently incubated with anti-phospho-FAK antibody (Tyr861, Cell Signaling Technology, MA, USA) and anti-β-actin antibody (Santa Cruz Biotechnology Inc., USA). Secondary probes were detected by ECL Western blot detection reagents (GE Healthcare, USA). And the images were captured and analyzed by a FluorChem 2 imaging system (Alpha Innotech, San Leandro, CA, USA).

### Statistical analysis

The results were expressed as means ± standard error of the mean (SEM). Multiple comparisons were performed using one-way ANOVA followed by LSD as a post-hoc test. Statistical differences between the two groups were analyzed by Student's t-test. A p-value less than 0.05 was selected as a criterion for a statistically significant difference.

## Results

**Figure 1:**
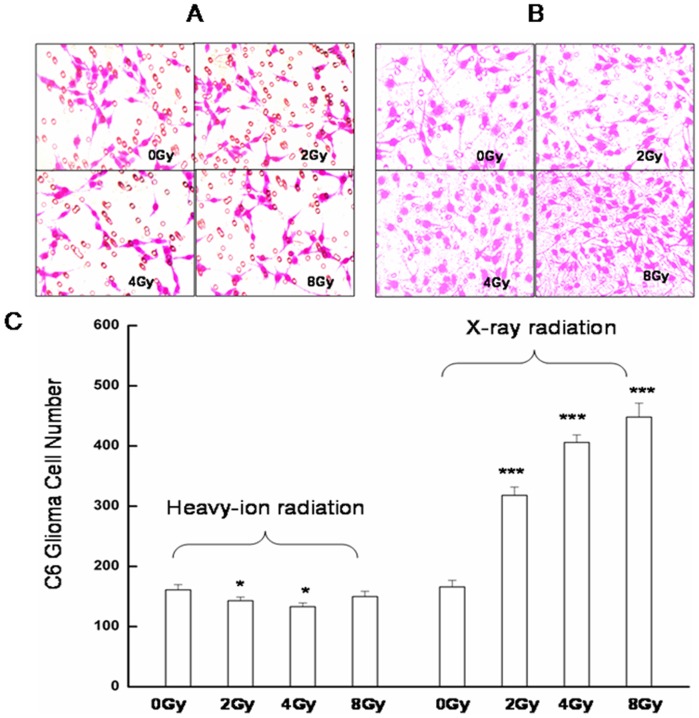
Effect of radiation on tumor cell migration as assessed by the Boyden chamber migration assay. Representative images are shown of migrating C6 glioma cells exposed to conditioned medium from cultures of **A**. unirradiated or heavy ion-irradiated, or **B**. unirradiated or X-ray-irradiated glioma cells. **C**. Quantification of the number of migratory glioma cells is expressed as means ± standard error of the mean from three independent experiments, in which treatments were performed in triplicate.*P<0.05, ***P<0.001 vs. controls (0 Gy).

Cancer cell migration and invasion into adjacent tissues, and intravasation into blood/lymphatic vessels are key events in malignant tumor progression. To investigate the effect of radiation on these processes, glioma and endothelial cells were treated with conditioned media from glioma cell cultures exposed to varying doses of radiation. Tumor-conditioned media from cultures exposed to carbon ion radiation suppressed migration by 11.8% at 2.0 Gy and 17.4% at 4.0 Gy compared to control cells treated with medium from unirradiated cultures after 24 h of exposure (Fig. 1A). In contrast, cell migration was markedly enhanced when media from X-ray-irradiated cultures was used as a chemoattractant (Fig. 1B).

**Figure 2:**
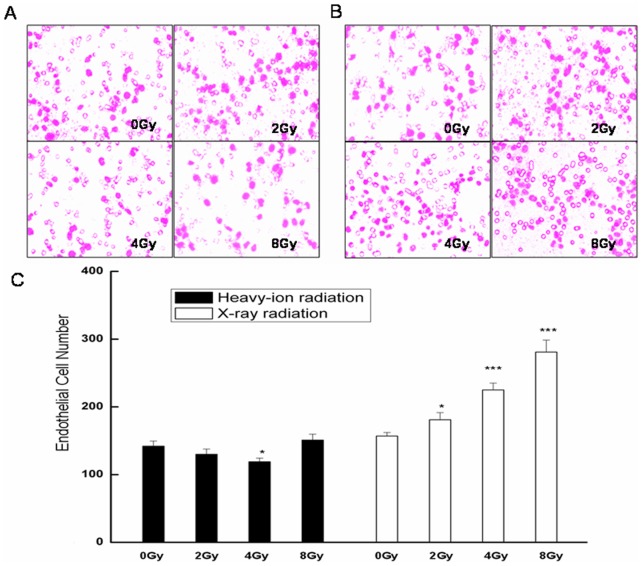
Effect of radiation on endothelial cell migration as assessed by the Boyden chamber migration assay. Representative images are shown of migrating HMEC-1 cells exposed to conditioned medium from cultures of **A**. unirradiated or heavy ion-irradiated, or **B**. unirradiated or X ray-irradiated glioma cells. **C**. Quantification of the number of migratory endothelial cells is expressed as means ± standard error of the mean from three independent experiments, in which treatments were performed in triplicate. *P<0.05, ^***^P<0.001 vs. controls (0 Gy).

Endothelial cell migration is essential for angiogenesis, a process that is closely associated with tumorigenesis and cancer progression. HMEC-1 cells incubated with conditioned media from carbon ion-irradiated cultures showed a decrease in migration at 4.0 Gy compared to controls (Fig. 2A, C), while in cells incubated with media from X-ray-irradiated cultures, a dose-dependent increase in migration was observed compared to control cells (Fig. 2B).

**Figure 3:**
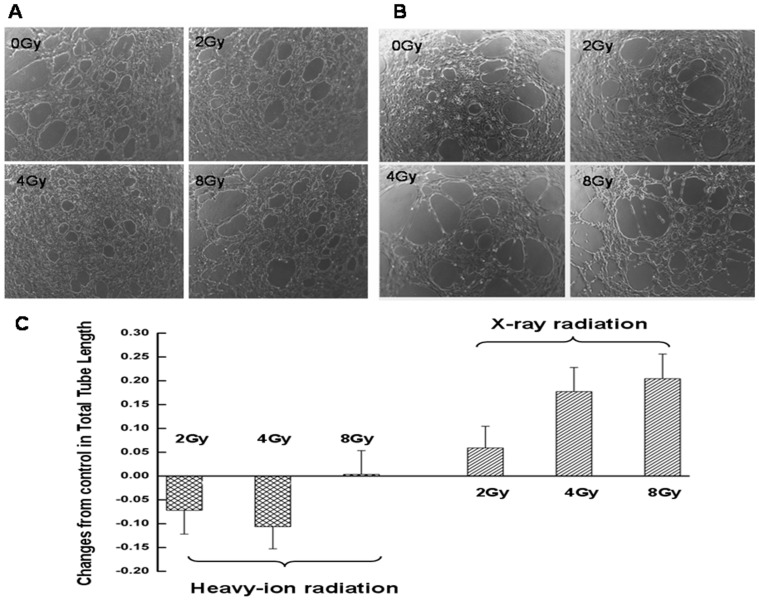
Effect of radiation on tube formation by endothelial cells as determined by the Matrigel assay. Representative images are shown of HMEC-1 cells cultured in Matrigel for 8 h in conditioned media from cultures of glioma cells exposed to **A**. carbon ion and **B**. X-ray radiation. **C**. Quantification of tube formation expressed as total tube length. Data represent the mean ± standard error of the mean of three independent experiments.

In later stages of angiogenesis, endothelial cells self-assemble into tubes to form new blood vessels. To investigate the effect of radiation on neovascularization, HMEC-1 cells were cultured on Matrigel-coated plates in the conditioned media. After an 8 h incubation, control cells formed tubular structures (Fig. 3A, B). Cells cultured in media from X-ray-irradiated glioma cell cultures formed a greater number of capillary-like structures than control cells (Fig. 3B); however, cells exposed to media from heavy ion-irradiated cells formed fewer tubules than controls (Fig. 3C).

**Figure 4:**
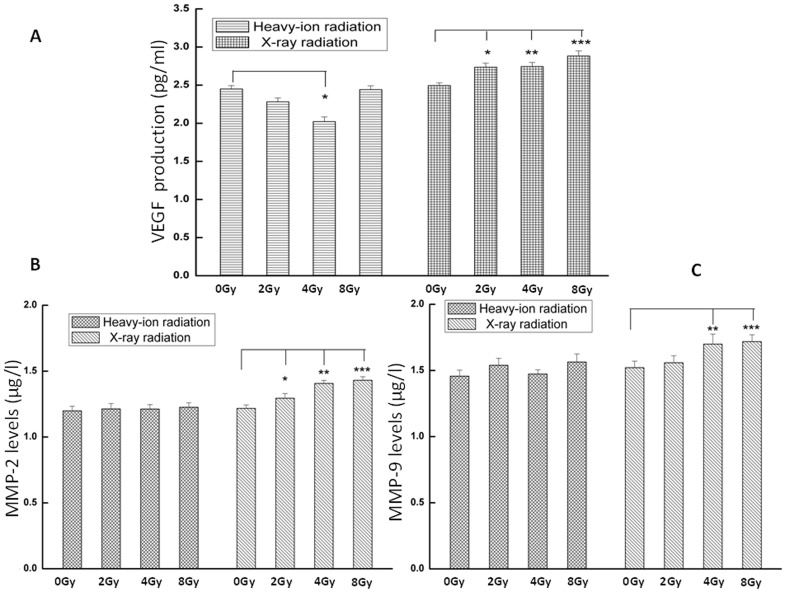
*VEGF* and *MMP*-*2* and -*9* protein concentration in conditioned media from cultures of irradiated tumor cells. C6 glioma cell culture supernatant was collected 24-ray irradiation, and protein levels were measured by ELISA. Data represent the mean ± standard error of the mean from six independent experiments. *P<0.05, **P<0.01, ***P<0.001 vs. controls (0 Gy).

*VEGF* and *MMP*-*2* and -*9* levels in conditioned media from cultures of glioma cells treated with varying doses of X-ray or carbon ion radiation were evaluated by ELISA. All three proteins were present at higher levels in the media of X-ray-irradiated cultures than in unirradiated cell culture medium (Fig. 4). There were no differences in the secretion of *MMP*-*2* and -*9* between carbon ion-irradiated cells and controls; however, *VEGF* concentration was 17.5% lower in the medium of cells irradiated with carbon ion at 4.0 Gy compared to the control culture (Fig. 4A).

**Figure 5:**
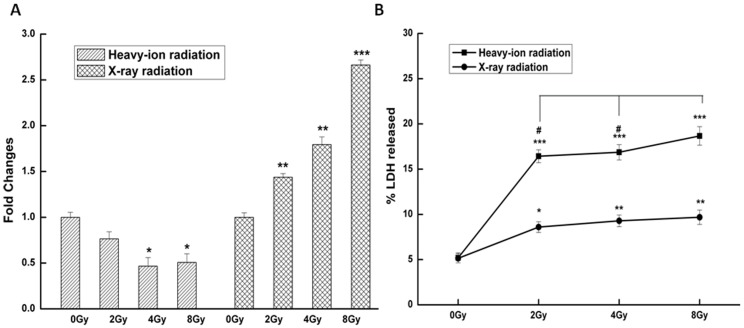
Effect of radiation on *VEGF* transcript expression and LDH activity. **A**. *VEGF* mRNA level and **B**. LDH activity were assessed in the culture medium of C6 glioma cells after treatment with carbon ion and X-ray radiation. Data represent the mean ± standard error of the mean from three replicates. *P<0.05, **P<0.01, ***P<0.001 vs. controls (0 Gy); ^#^P<0.05 vs. 8.0 Gy group.

To determine whether the altered levels of *VEGF* protein in tumor-conditioned media were due to transcriptional upregulation, the *VEGF* mRNA level in glioma cells was assessed by RT-qPCR 24 h after irradiation. *VEGF* transcript levels in the 2.0, 4.0, and 8.0 Gy X-ray radiation groups were higher by approximately 1.44-, 1.79-, and 2.66-fold relative to the control group (Fig. 5A). In contrast, exposure to carbon ion radiation resulted in the downregulation of *VEGF* gene expression at 4.0 (0.46±0.09) and 8.0 Gy (0.48±0.09). LDH is a sensitive indicator of the metabolic state of cells, and is rapidly released into the culture medium upon damage to the plasma membrane. Irradiated glioma cells had higher LDH activity in the medium compared to unirradiated control cells (Fig. 5B). Notably, cells exposed to 8.0 Gy carbon ion radiation had increased LDH in the medium compared to those exposed to the lower doses of 2.0 and 4.0 Gy, indicating that higher carbon ion radiation doses produced greater plasma membrane damage.

**Figure 6:**
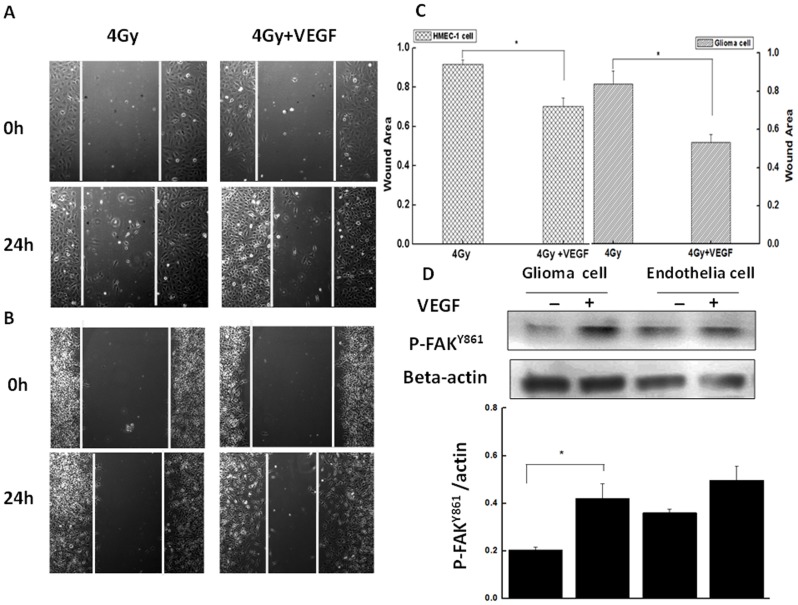
*VEGF*-induced endothelial and glioma cell migration. **A**. HMEC-1 and **B**. C6 glioma cells were cultured in media from cultures of carbon ion-irradiated glioma cells supplemented with *VEGF165* (50 ng/ml). **C**. Quantification of migrating cells. Data represent the mean ± standard error of the mean from at least three independent experiments. **D**. Expression of phosphorylated FAK (Y861) in cells after addition of *VEGF165*, as determined by western blotting.

To examine the functional significance of altered *VEGF* level in the culture medium of irradiated tumor cells, conditioned media were supplemented with recombinant *VEGF165* (50 ng/ml) and the effect on cell migration was evaluated by wound-healing assay. The migration of HMEC-1 and C6 glioma cells was increased by 1.31- and 1.58-fold, respectively, in the presence of *VEGF165* (Fig. 6C). Moreover, after a 4 h incubation in media from carbon ion-irradiated cell cultures supplemented with *VEGF165*, both cell types showed increases in levels of phosphorylated FAK (Y861) (Fig. 6D).

## Discussion

Radiotherapy with or without surgery is a standard treatment for malignant gliomas [14]. However, tumor cell responses to radiation are complex, with evidence suggesting that radiation from X- and γ-rays not only cause the death of tumor cells, but can alternatively lead to the emergence of greater aggressivity and malignancy, allowing cells to escape the lethal effects of radiation and remain resistant to conventional therapy [15]–[16].

Heavy ion radiation therapy has shown promise for the treatment of head and neck cancers—even those that are resistant to standard radiation by directly targeting the DNA of tumor cells [17]–[18]. In the present study, the effects of high LET carbon ion and low LET X-ray radiation on glioma and endothelial cell migration were compared, using tumor-conditioned medium as an inducer. Media from cultures of C6 glioma cells exposed to 2.0 or 4.0 Gy carbon ion radiation suppressed migration of both cell types (Figs. 1 and 2), suggesting that irradiation with heavy ions can potentially attenuate the growth and metastasis of glioblastomas. In addition, the formation of endothelial cell tubules was inhibited upon incubation with the same conditioned medium (Fig. 3), implying that angiogenesis can be similarly blocked. Carbon ion radiation has been shown to suppress migration and the formation of capillary-like tube structures in ECV304 and human umbilical vascular endothelial cells at a dose of 0.1 Gy [19]. In contrast, conditioned media from tumor cells exposed to X-ray radiation stimulated cell migration and tube formation, consistent with previous findings [20]. The migration and invasion of glioma cells into surrounding normal brain tissue and blood vessels contributes to the limited responsiveness of gliomas to conventional radiation treatments [21]–[22]. Thus, for resistant tumors, carbon ions can offer greater therapeutic effectiveness compared to X-rays owing to the superior physical properties of high LET particles that transfer a greater amount of energy per unit length of track, which consequently increases the spatial density of energy deposition events [23].

The tumor microenvironment is dynamic and can be altered by radiation treatment. In accordance with earlier studies [20], [24]–[25], the present data showed that X-ray radiation enhanced the secretion of *VEGF*, a potent angiogenic growth factor [26], as well as that of *MMP*-*2* and -*9*, which degrade the basement membrane and extracellular matrix during tumor cell invasion and metastasis [27]. In contrast, *VEGF* and *MMP*-*2* and -*9* levels were not significantly increased by carbon ion radiation; indeed, *VEGF* secretion was reduced at 4.0 Gy (Fig. 4), implying that carbon ions may suppress glioblastoma angiogenicity and tumorigenicity by modulating *VEGF* production and release into the tumor microenvironment. However, the secreted *VEGF* induced by carbon ion radiation was not dose-dependent, and reached a minimum at 4.0 Gy. One explanation for this observation is that the expression of pro-angiogenic genes and disruption of tumor cell membrane integrity are largely determined by growth factor secretion. Thus, although *VEGF* transcript expression was suppressed at 4.0 and 8.0 Gy carbon ion radiation, the increase in the permeability of the cell membrane as reflected by LDH activity in the culture medium was more obvious at 8.0 Gy, because damage to the plasma membrane at the higher radiation dose resulted in the release of proteins into the medium. Therefore, in view of the combined effects of above-mentioned factors, the minimum production of *VEGF* in the conditioned medium was exhibited at 4.0 Gy carbon-ion radiation group.

To identify the mechanism by which carbon ion radiation-induced *VEGF* stimulates cell motility, cells were treated with recombinant *VEGF165* and the phosphorylation status of the downstream factor FAK was examined. FAK is a non-receptor tyrosine kinase present at cell-cell contacts that is involved in integrin-mediated signal transduction [28], and tyrosine phosphorylation of FAK is required for cell migration [29]–[30]; *VEGF* induces FAK phosphorylation at Y397, Y407, and Y861 [31]–[32]. Here, the application of exogenous *VEGF* increased migration in endothelial and glioma cells concomitant with increased FAK phosphorylation at Y861 (Fig. 6), indicating that carbon ion radiation may promote the metastatic and angiogenic potential of tumors through *VEGF*/FAK signaling.

In summary, the present results demonstrate that high-LET carbon ion radiation is superior to low-LET X-rays for suppressing the migration of glioma and endothelial cells, and thus inhibiting tumorigenesis and angiogenesis via regulation of *VEGF*-mediated signaling in the tumor microenvironment. These findings provide evidence in favor of carbon ion over X-ray radiation for the treatment of tumors that are resistant to conventional radiotherapy, as well as insight into possible *in vivo* effects of carbon ion radiation on the tumor microenvironment, which can inform the development of more effective therapeutic strategies that can improve disease outcome.
