# Correction: Carbon Ion Radiation Inhibits Glioma and Endothelial Cell Migration Induced by Secreted VEGF

**DOI:** 10.1371/journal.pone.0135508

**Published:** 2015-08-07

**Authors:** Yang Liu, Yuanyuan Liu, Chao Sun, Lu Gan, Luwei Zhang, Aihong Mao, Yuting Du, Rong Zhou, Hong Zhang

Concerns were raised by a reader about Fig 1A and 2A of this paper, where multiple panels appear to have been derived from the same microscopic image.

PLOS Staff Editors contacted the authors for a response to these concerns and to request the original images for these figures. The authors acknowledged that some of the panels were derived from the same image, which they suggested was caused by errors when cropping and cutting the original images.

The authors have declared that the figure errors have no effect on the results and conclusions of the study. They have provided the journal office with the original and corrected images for Figs 1 and 2. The original images and corrected figures have been reviewed by a Section Editor, who is satisfied with the authors' response to the concerns.

Please view the corrected images for Figs 1 and 2 below. The original images are included in S1 File.

**Fig 1:**
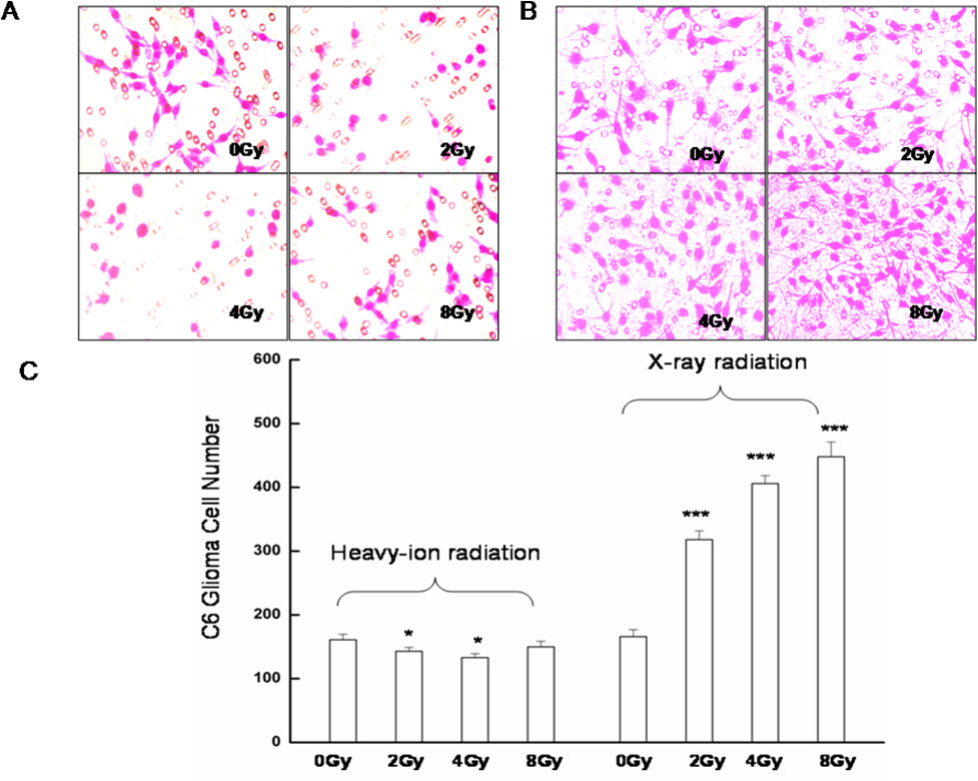
Effect of radiation on tumor cell migration as assessed by the Boyden chamber migration assay. Representative images are shown of migrating C6 glioma cells exposed to conditioned medium from cultures of A. unirradiated or heavy ion-irradiated, or B. unirradiated or X-ray-irradiated glioma cells. C. Quantification of the number of migratory glioma cells is expressed as means ± standard error of the mean from three independent experiments, in which treatments were performed in triplicate.*P<0.05, ***P<0.001 vs. controls (0 Gy).

**Fig 2:**
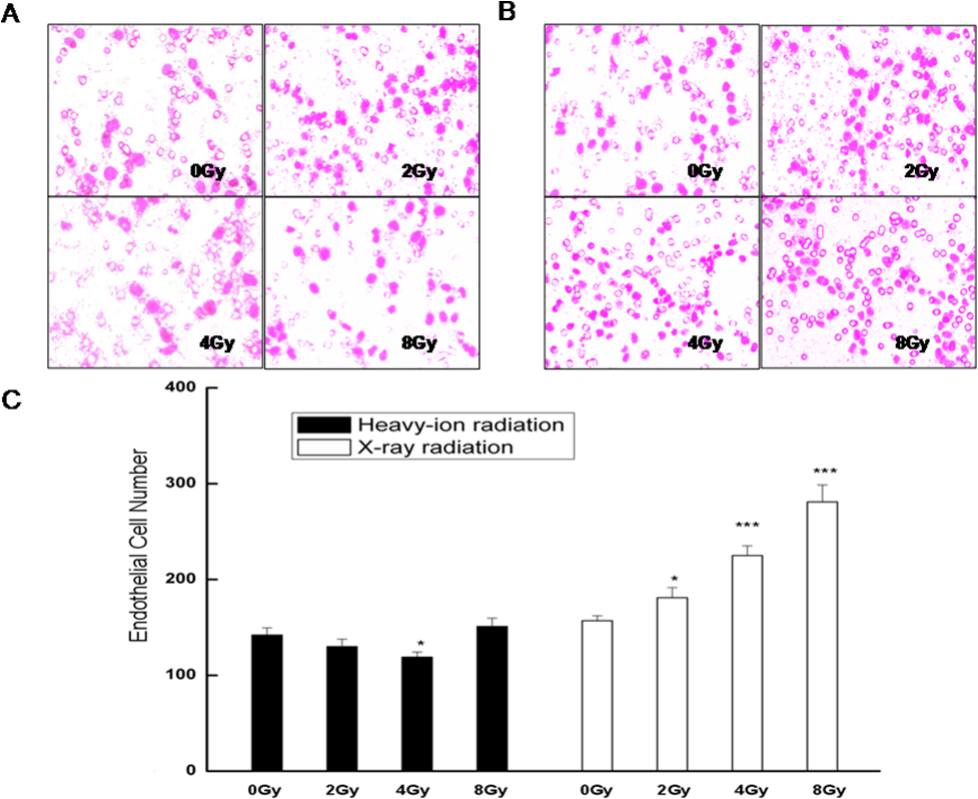
Effect of radiation on endothelial cell migration as assessed by the Boyden chamber migration assay. Representative images are shown of migrating HMEC-1 cells exposed to conditioned medium from cultures of A. unirradiated or heavy ion-irradiated, or B. unirradiated or X ray-irradiated glioma cells. C. Quantification of the number of migratory endothelial cells is expressed as means ± standard error of the mean from three independent experiments, in which treatments were performed in triplicate. *P<0.05, ***P<0.001 vs. controls (0 Gy).

## Supporting Information

S1 File Original images for Fig 1A and Fig 2A. (ZIP)

## References

[1] Liu Y, Liu Y, Sun C, Gan L, Zhang L, Mao A, et al (2014) Carbon Ion Radiation Inhibits Glioma and Endothelial Cell Migration Induced by Secreted VEGF. PLoS ONE 9(6): e98448 doi:10.1371/journal.pone.0098448 24893038 10.1371/journal.pone.0098448 PMC4043910

