# Supplementary figures and images for: Correction: Carbon Ion Radiation Inhibits Glioma and Endothelial Cell Migration Induced by Secreted VEGF
Source: PLoS One. 2015 Aug 7;10(8):e0135508. doi: 10.1371/journal.pone.0135508 (PMC4529295; doi:10.1371/journal.pone.0135508)

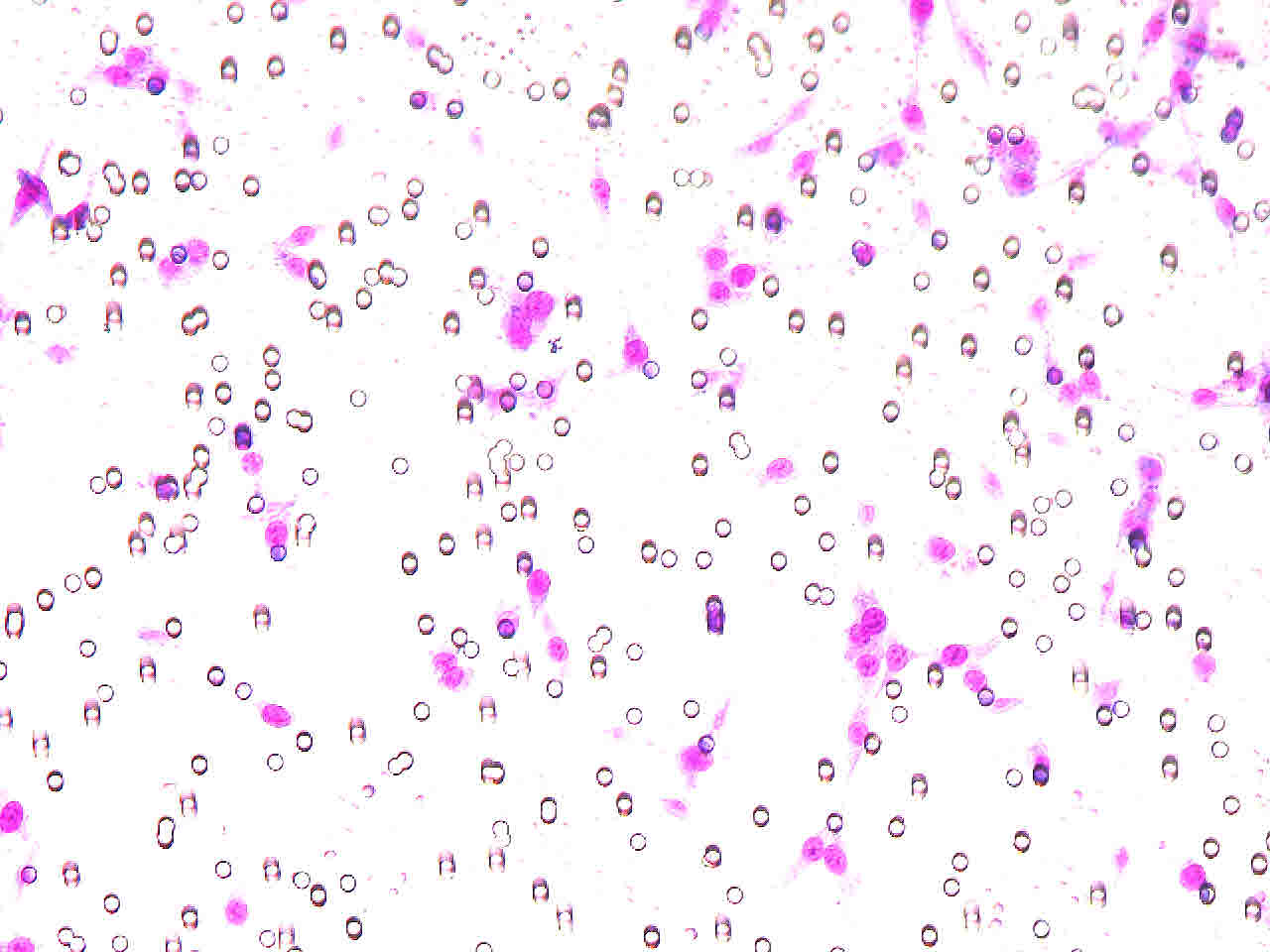

Supplement: S1 File — (ZIP) [file pone.0135508.s001.zip › figure1a/Figure1A-3/4Gy-3.jpg]

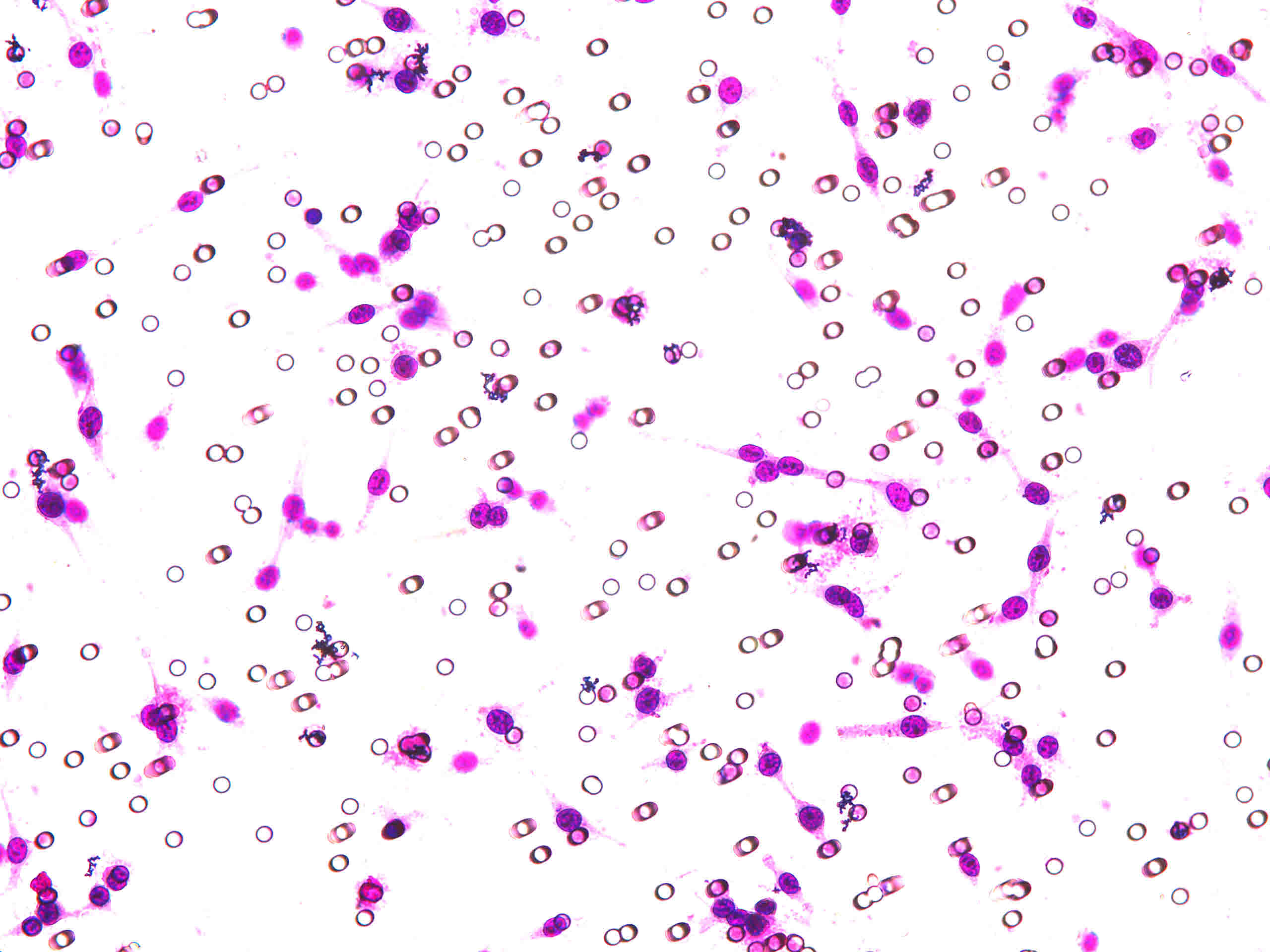

Supplement: S1 File — (ZIP) [file pone.0135508.s001.zip › figure1a/Figure1A-3/2Gy-1.jpg]

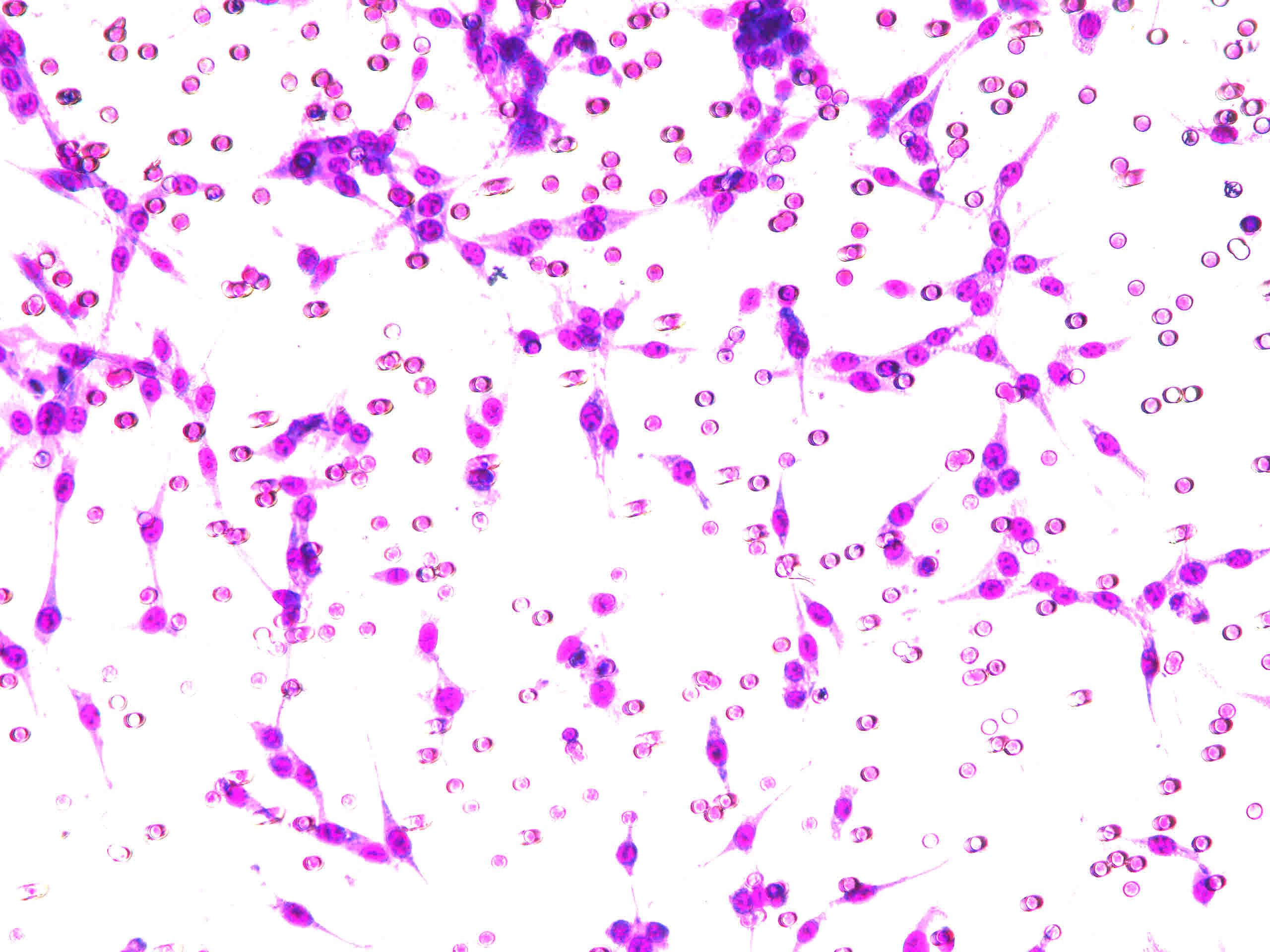

Supplement: S1 File — (ZIP) [file pone.0135508.s001.zip › figure1a/Figure1A-3/0Gy-1.jpg]

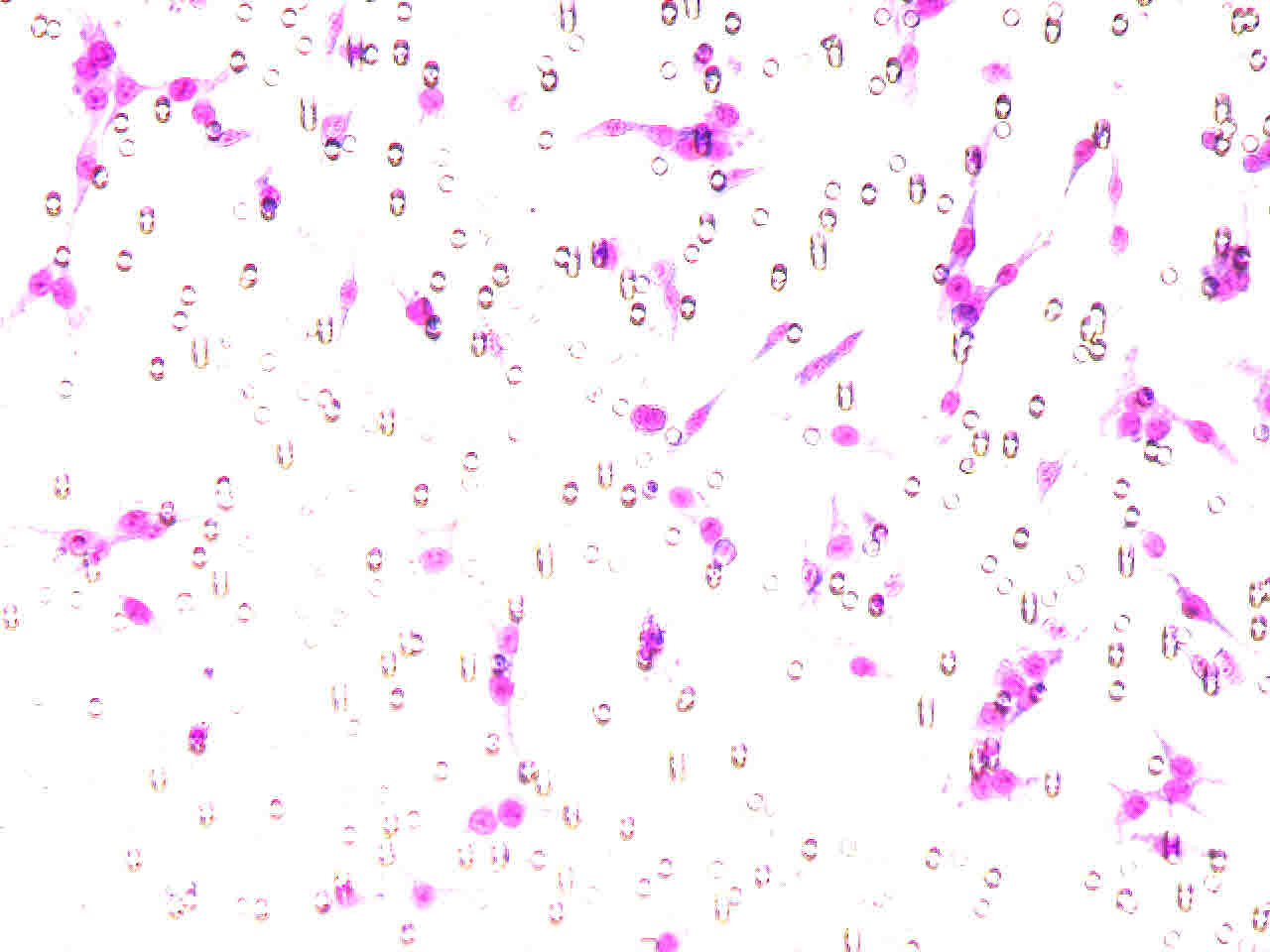

Supplement: S1 File — (ZIP) [file pone.0135508.s001.zip › figure1a/Figure1A-3/4Gy-2.jpg]

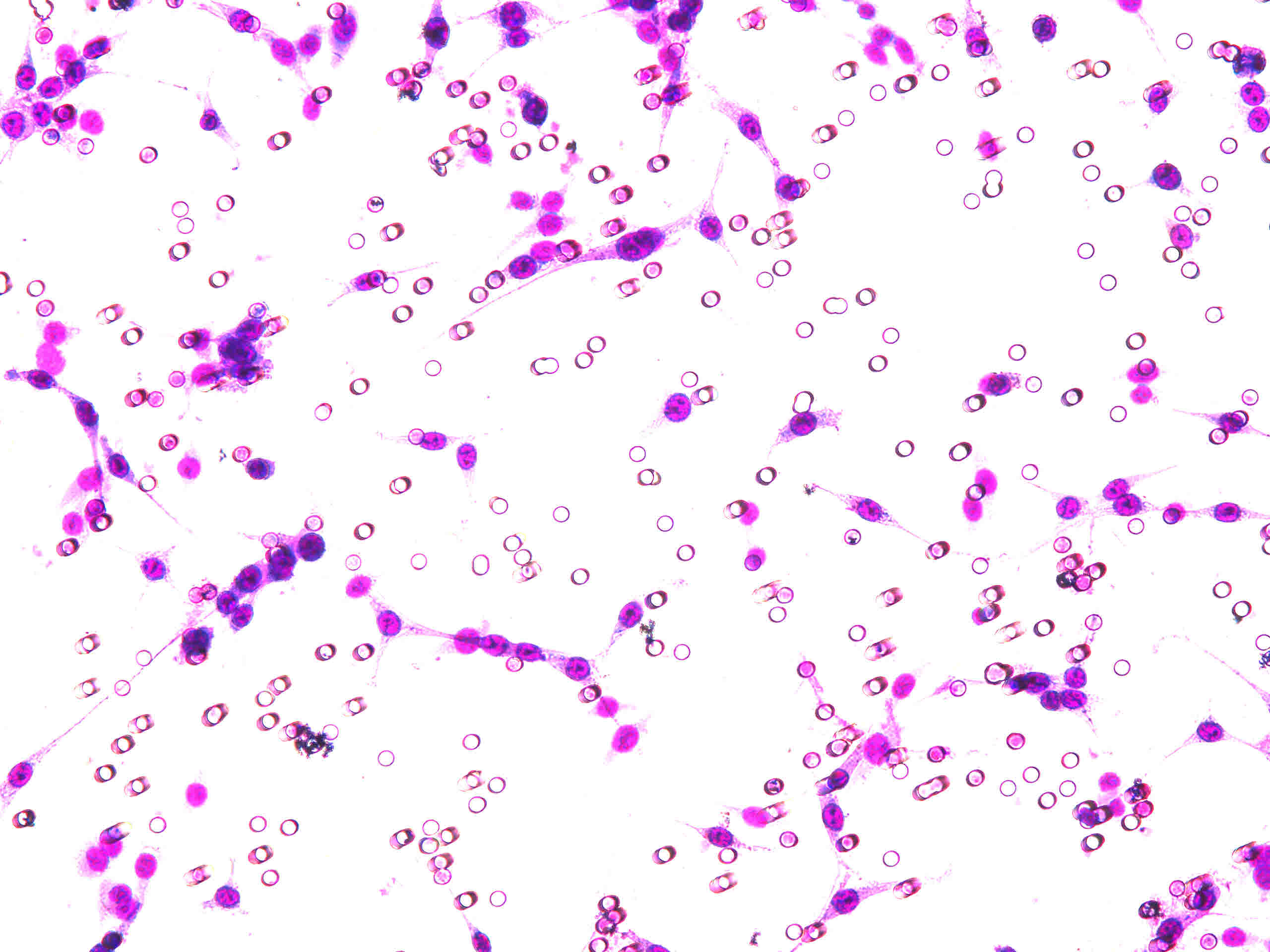

Supplement: S1 File — (ZIP) [file pone.0135508.s001.zip › figure1a/Figure1A-3/2Gy-2.jpg]

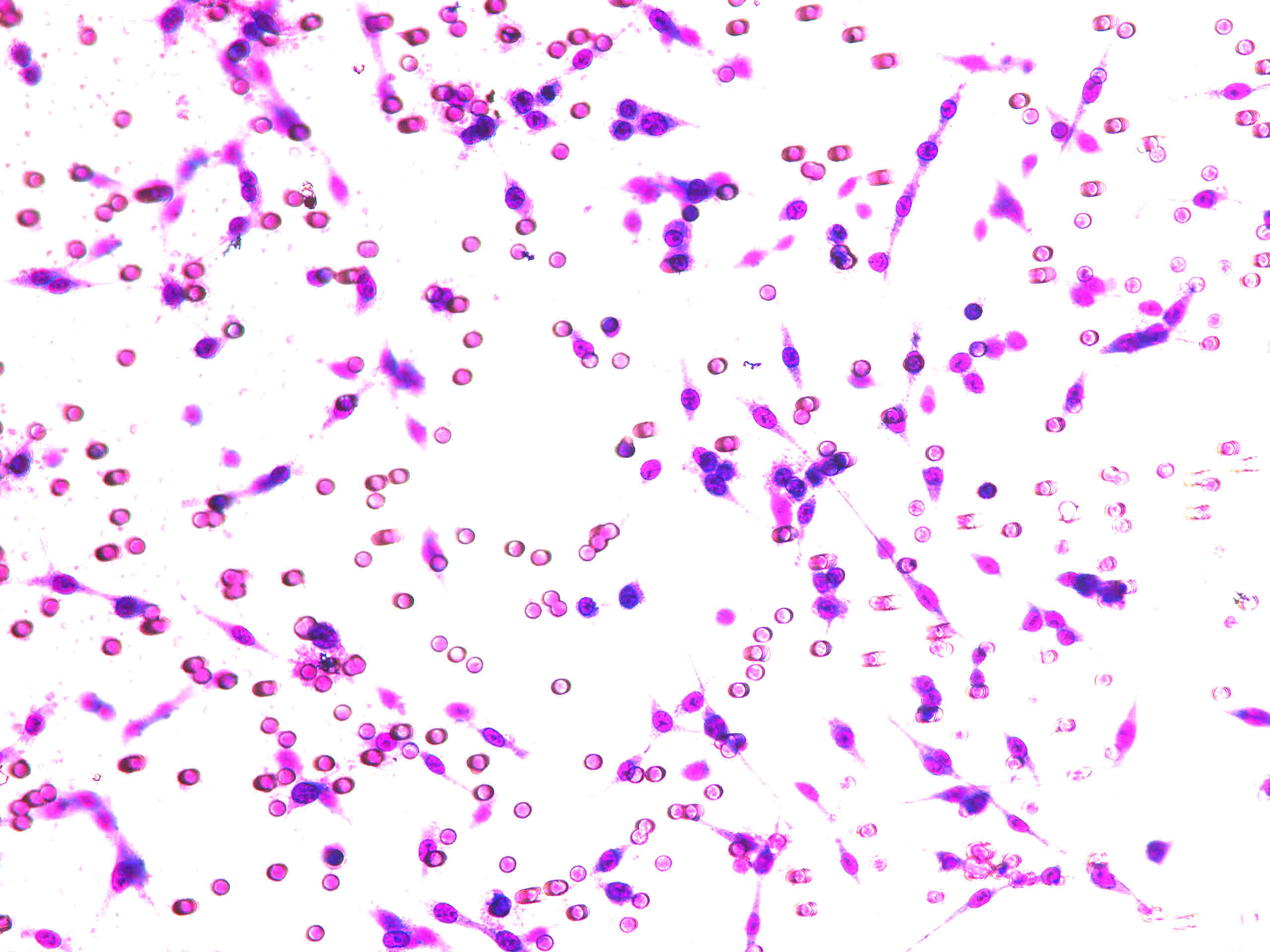

Supplement: S1 File — (ZIP) [file pone.0135508.s001.zip › figure1a/Figure1A-3/8Gy-4.jpg]

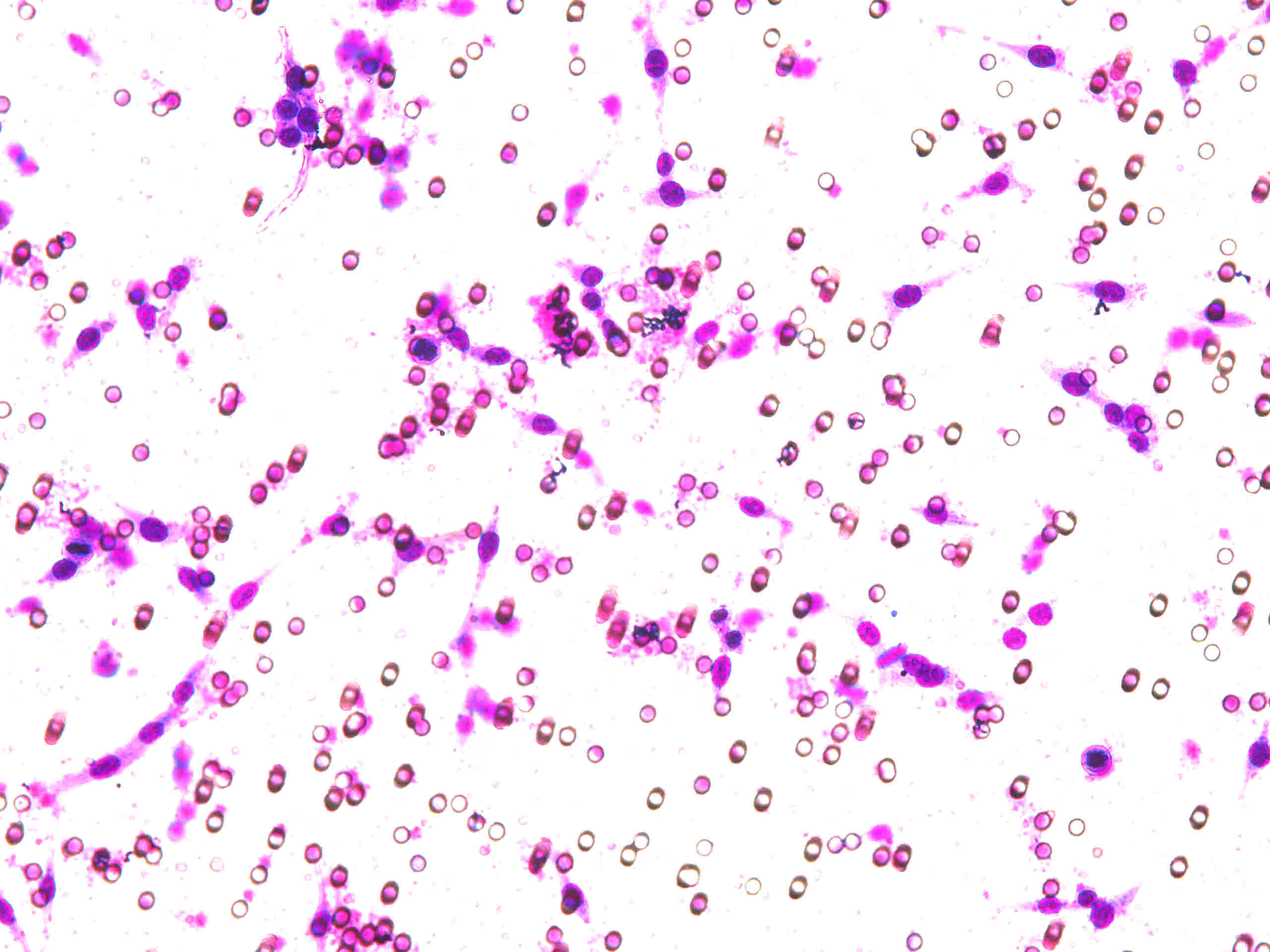

Supplement: S1 File — (ZIP) [file pone.0135508.s001.zip › figure1a/Figure1A-3/0Gy-3.jpg]

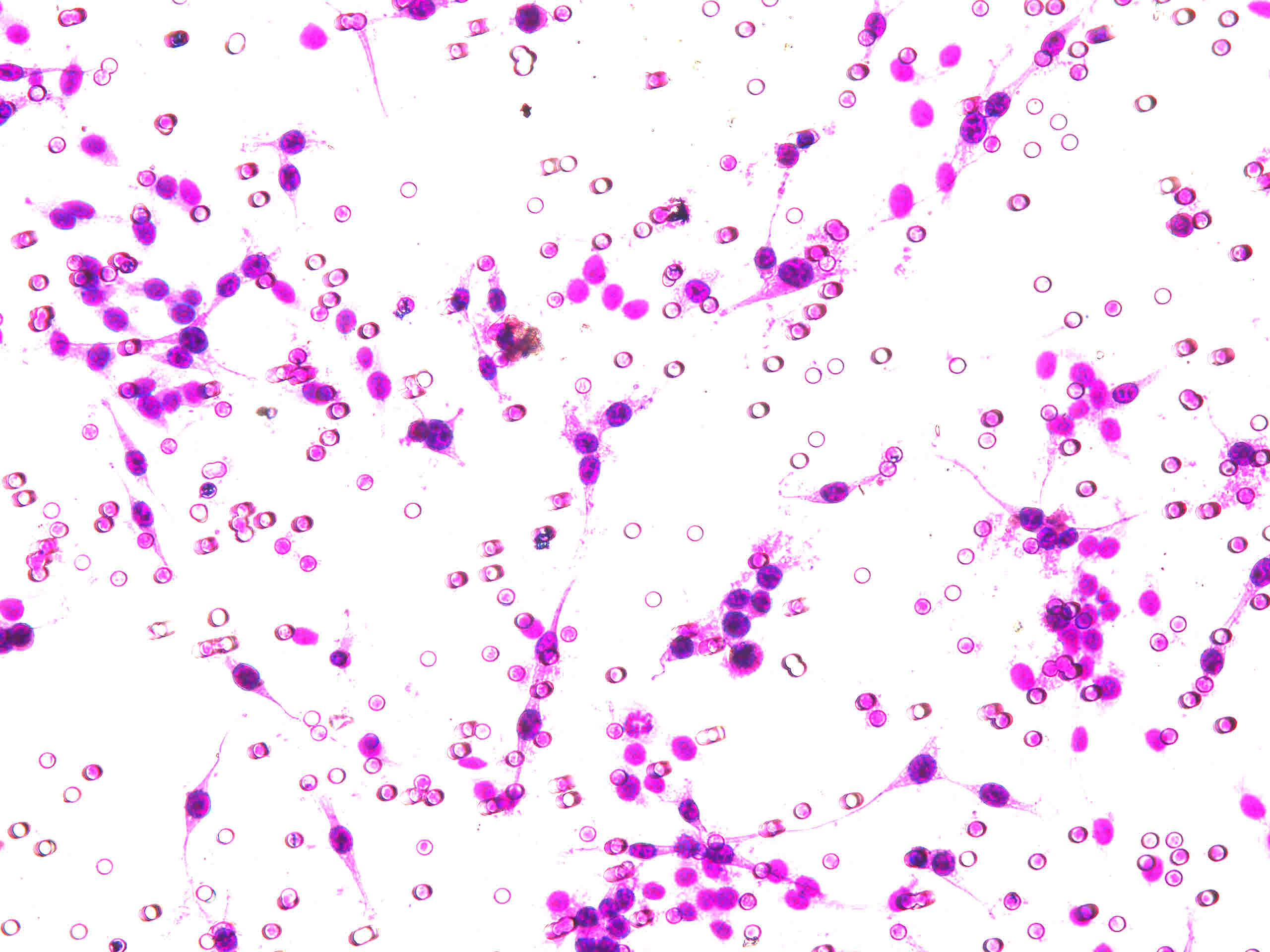

Supplement: S1 File — (ZIP) [file pone.0135508.s001.zip › figure1a/Figure1A-3/0Gy-2.jpg]

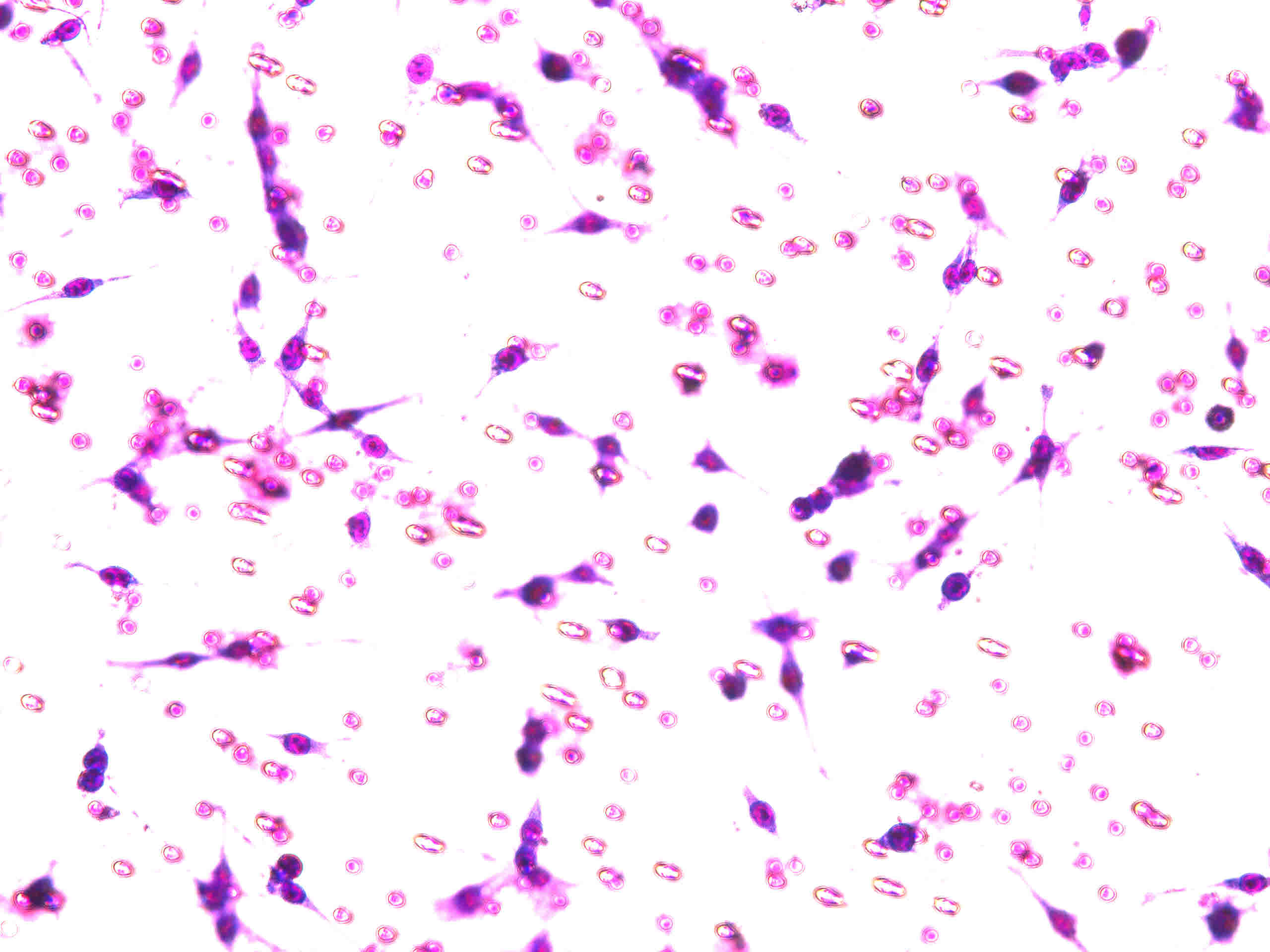

Supplement: S1 File — (ZIP) [file pone.0135508.s001.zip › figure1a/Figure1A-3/2Gy-3.jpg]

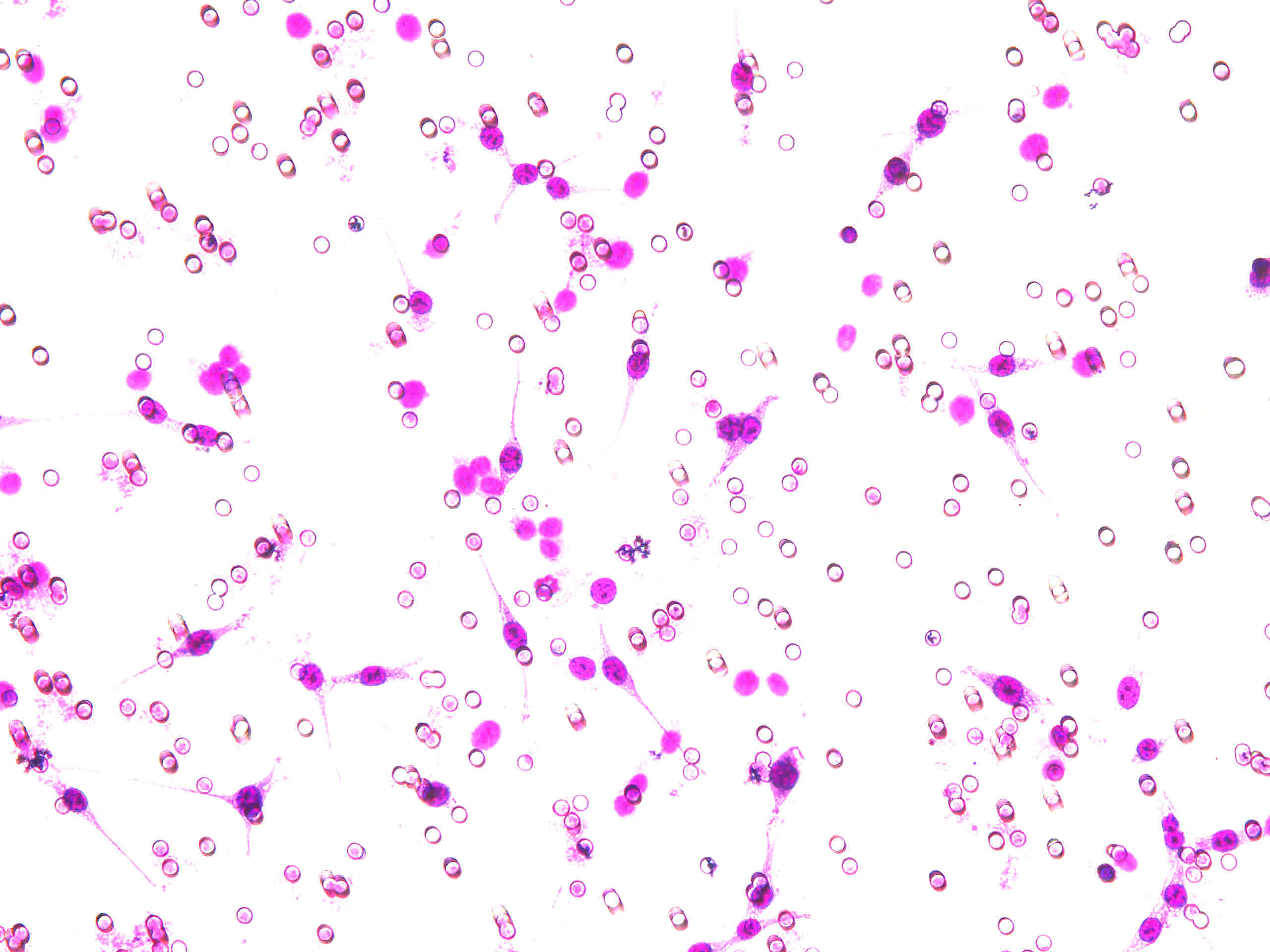

Supplement: S1 File — (ZIP) [file pone.0135508.s001.zip › figure1a/Figure1A-3/4Gy-1.jpg]

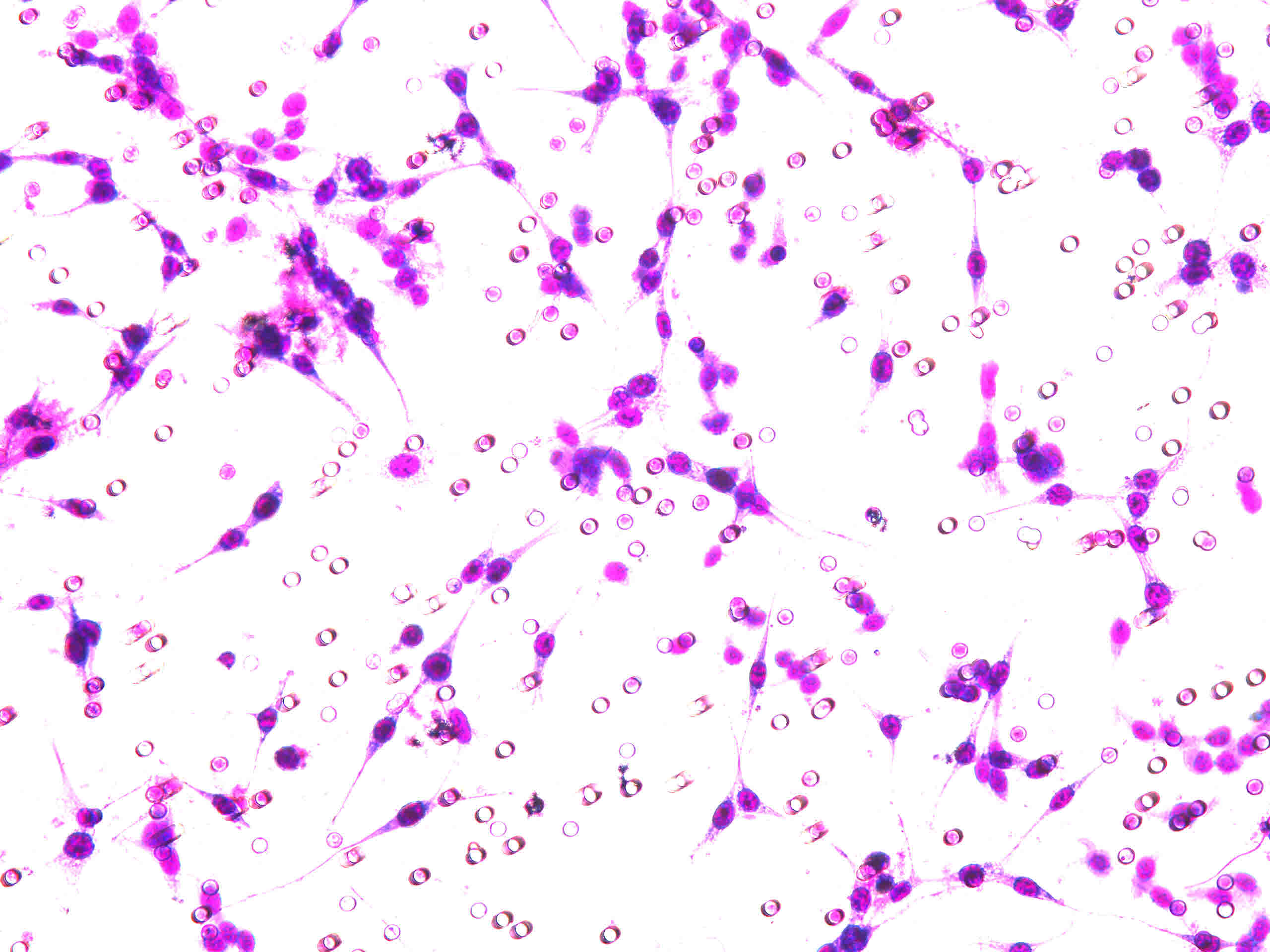

Supplement: S1 File — (ZIP) [file pone.0135508.s001.zip › figure1a/Figure1A-3/8Gy-1.jpg]

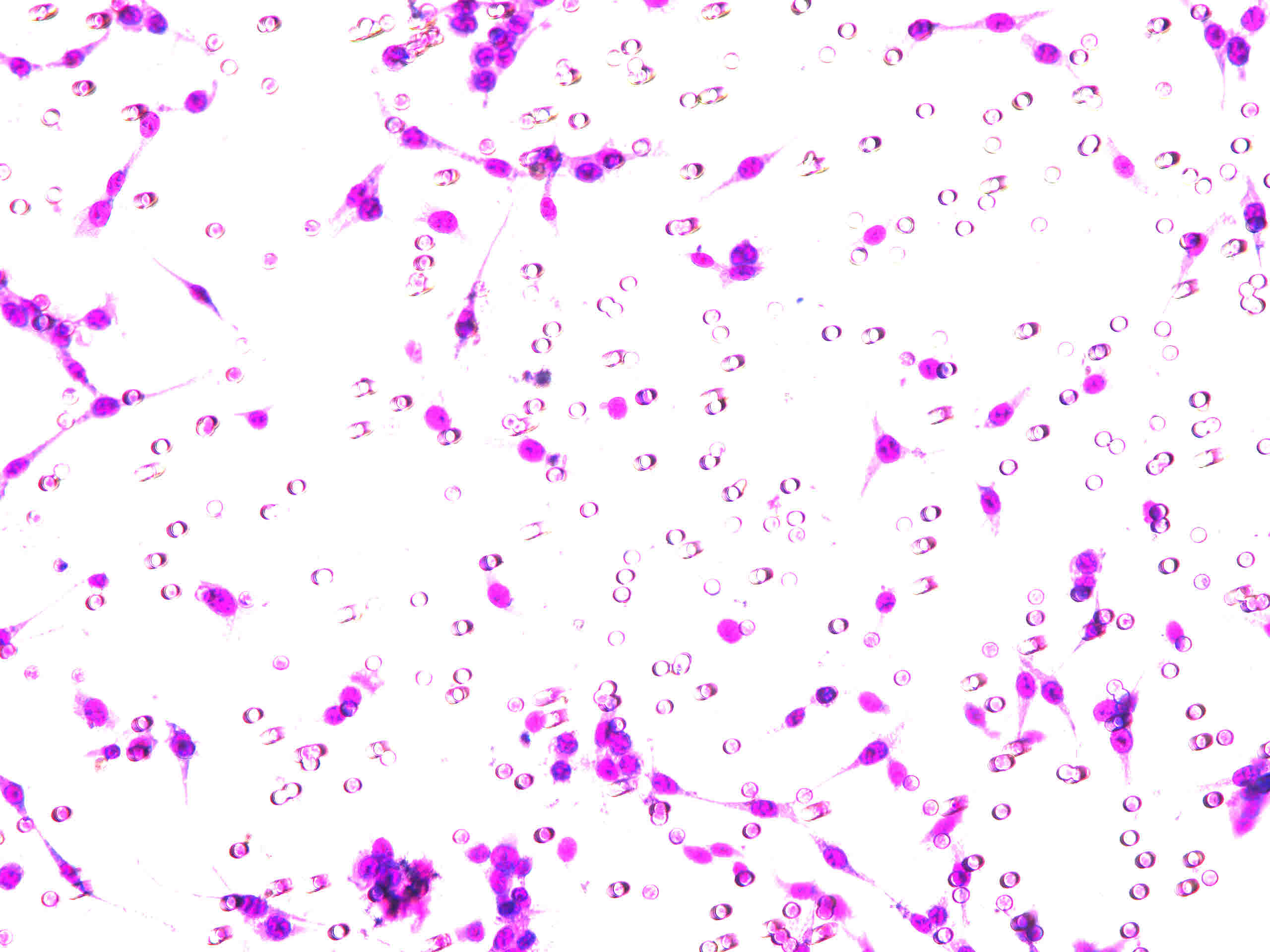

Supplement: S1 File — (ZIP) [file pone.0135508.s001.zip › figure1a/Figure1A-3/4Gy-4.jpg]

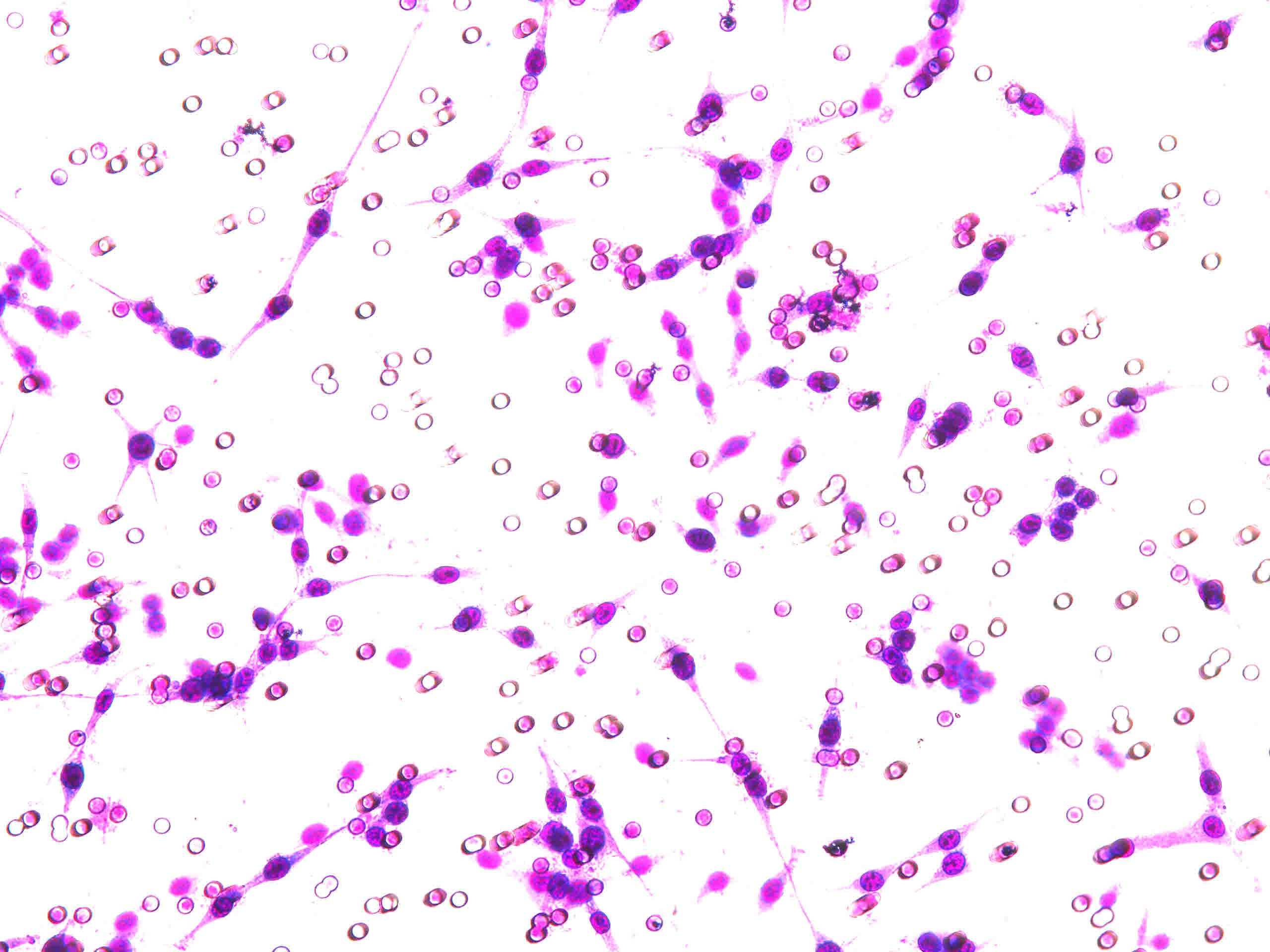

Supplement: S1 File — (ZIP) [file pone.0135508.s001.zip › figure1a/Figure1A-3/8Gy-2.jpg]

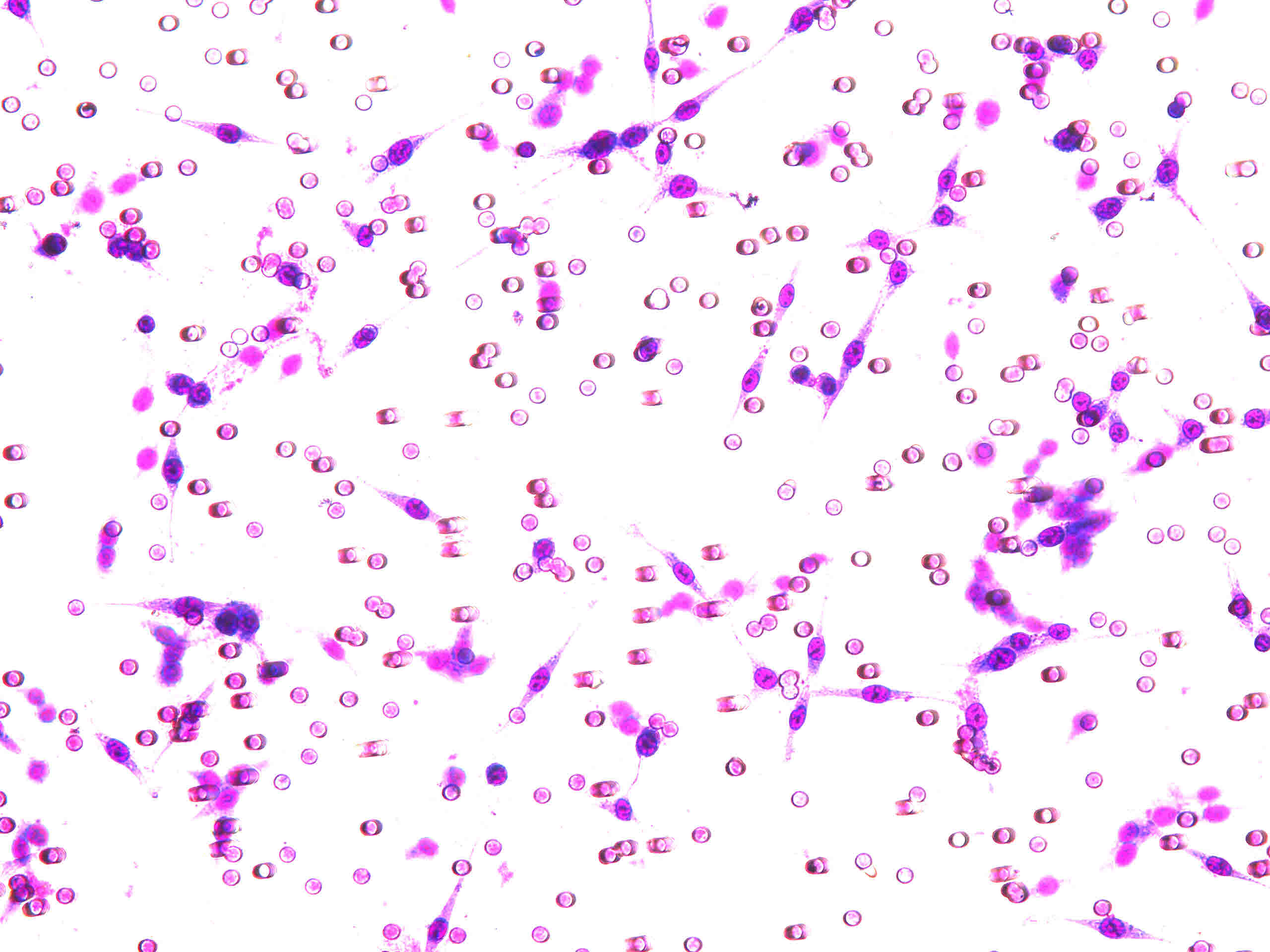

Supplement: S1 File — (ZIP) [file pone.0135508.s001.zip › figure1a/Figure1A-3/8Gy-3.jpg]

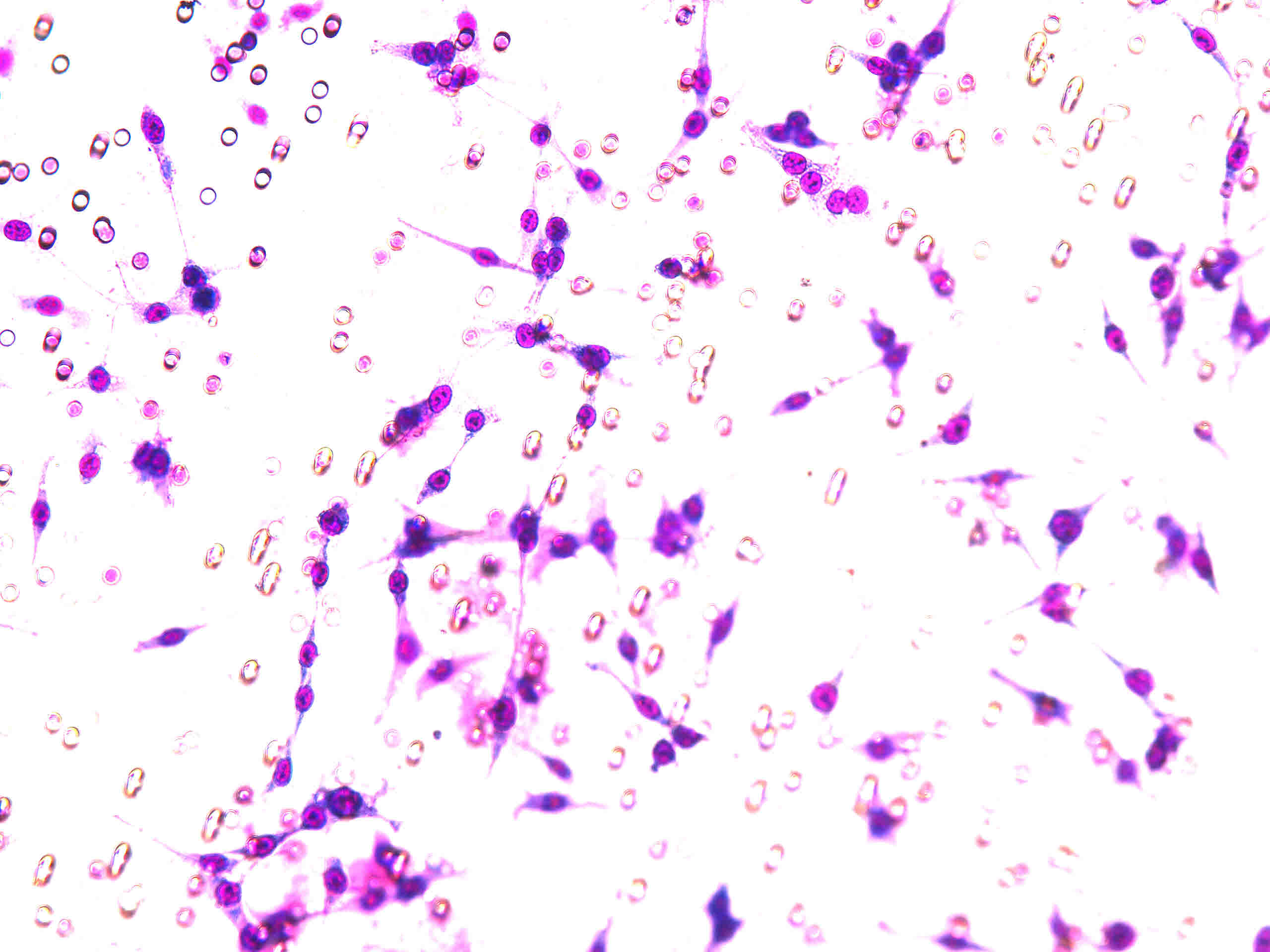

Supplement: S1 File — (ZIP) [file pone.0135508.s001.zip › figure1a/Figure1A-3/0Gy-4.jpg]

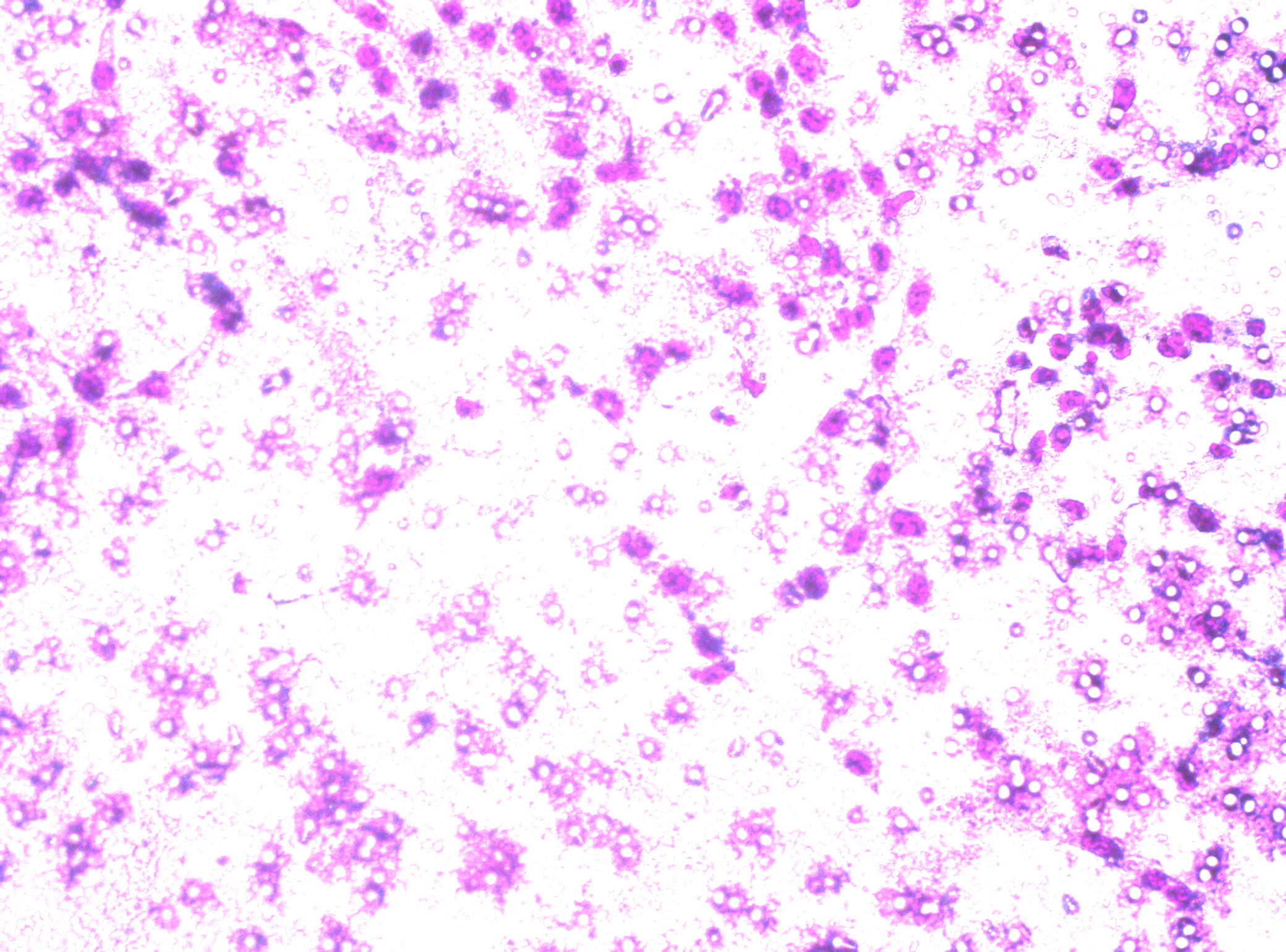

Supplement: S1 File — (ZIP) [file pone.0135508.s001.zip › figure2a/Figure2A-1/4Gy-1.jpg]

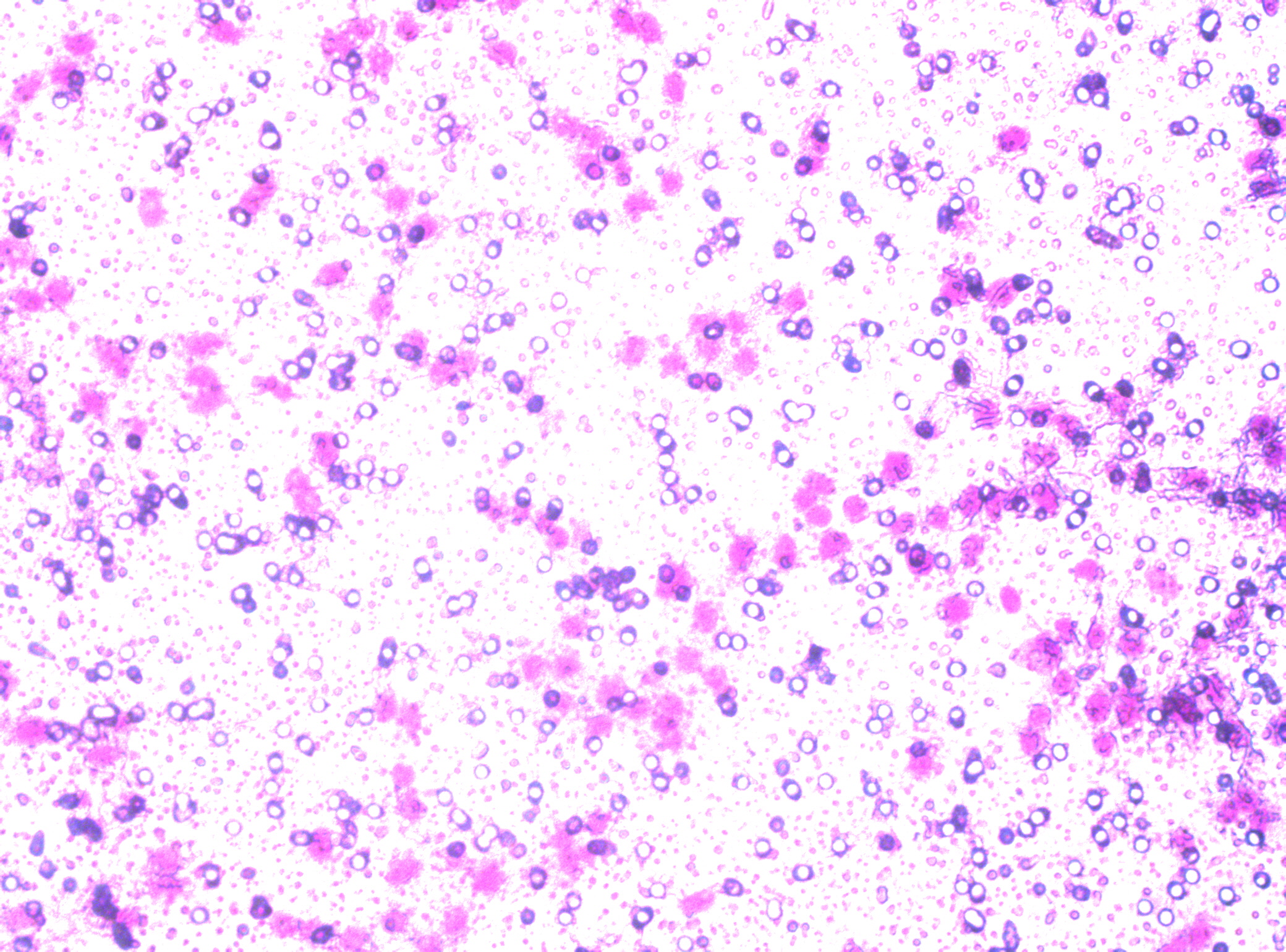

Supplement: S1 File — (ZIP) [file pone.0135508.s001.zip › figure2a/Figure2A-1/2Gy-1.jpg]

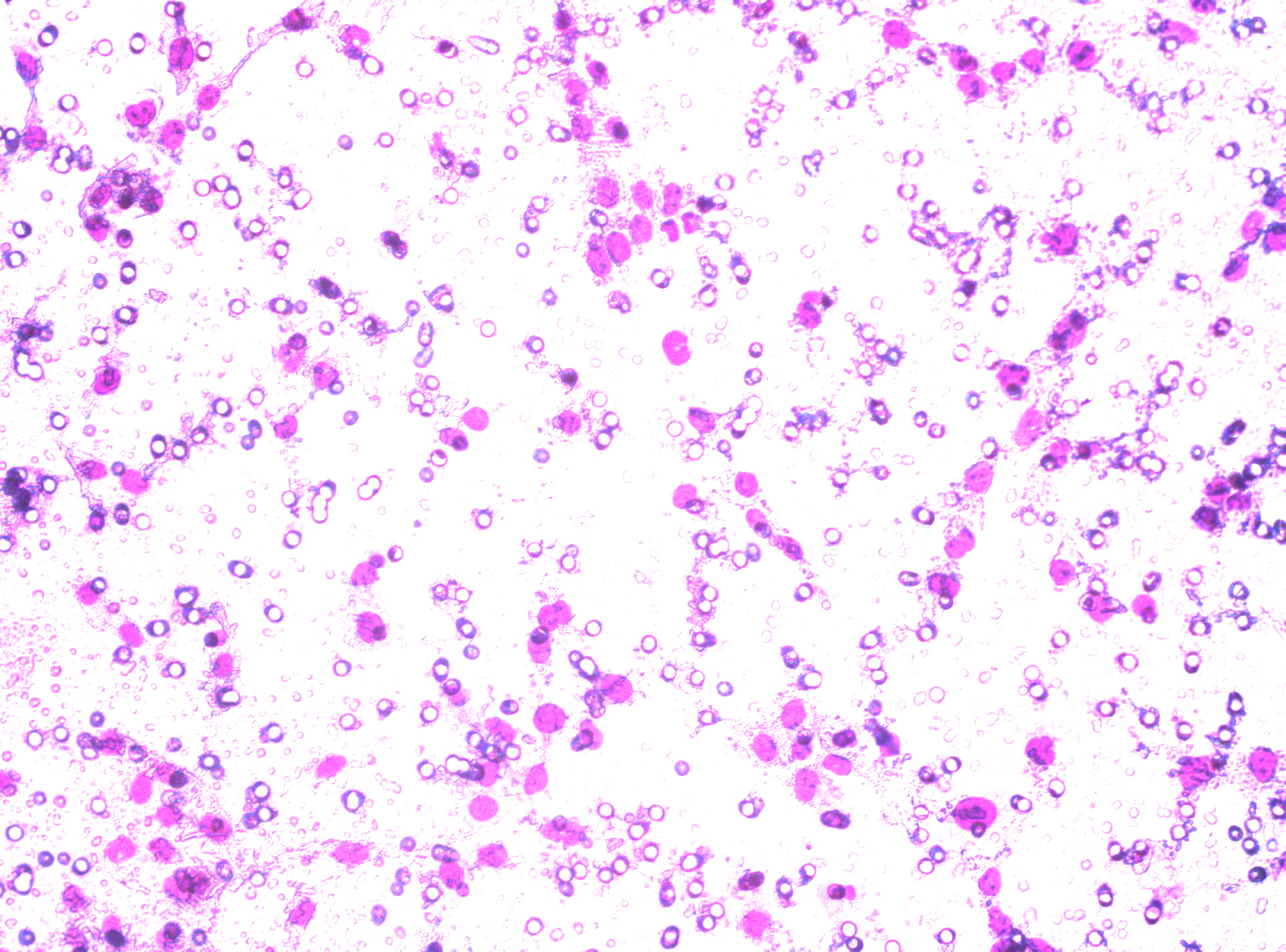

Supplement: S1 File — (ZIP) [file pone.0135508.s001.zip › figure2a/Figure2A-1/0Gy-1.jpg]

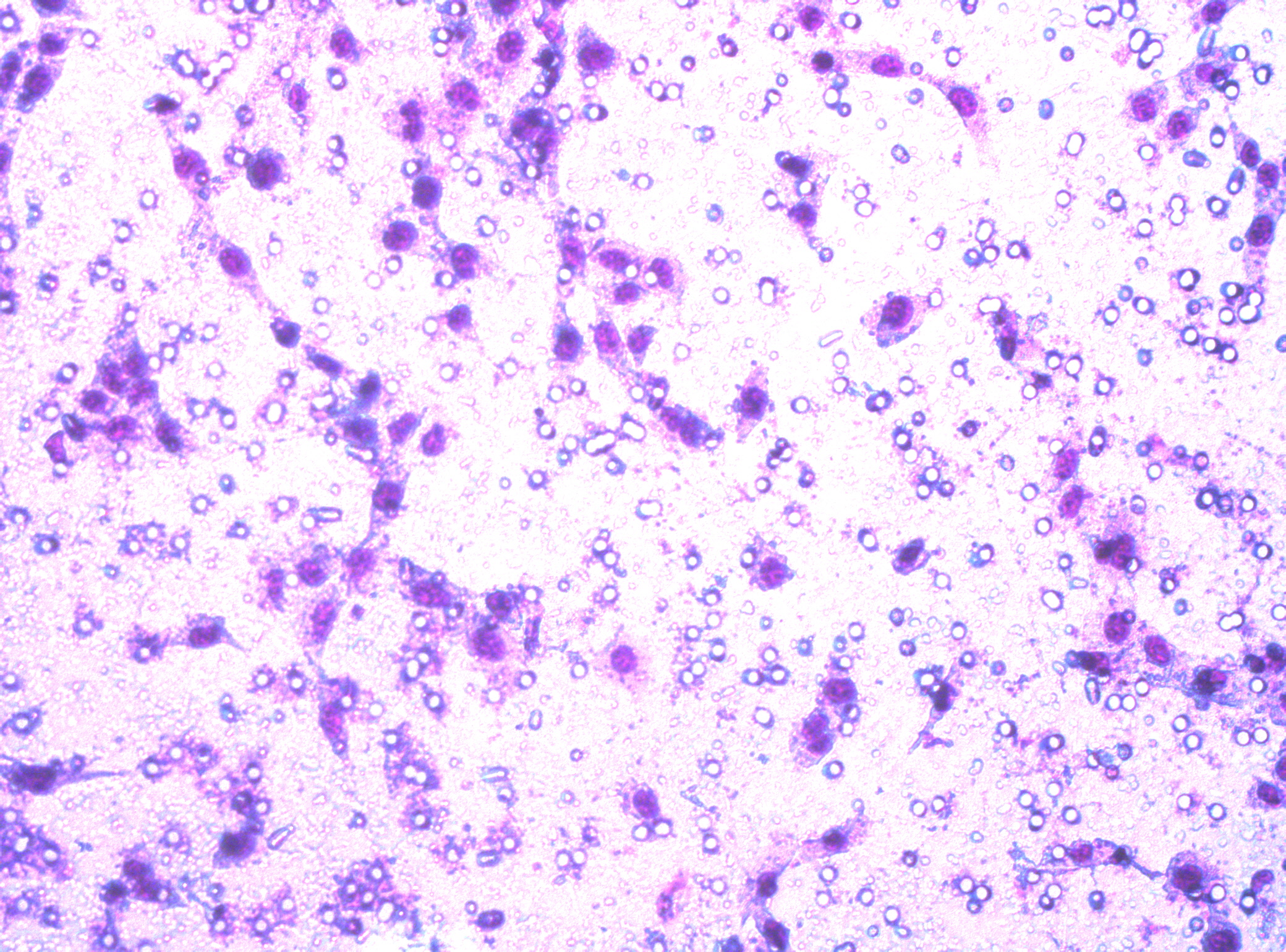

Supplement: S1 File — (ZIP) [file pone.0135508.s001.zip › figure2a/Figure2A-1/8Gy-1.jpg]

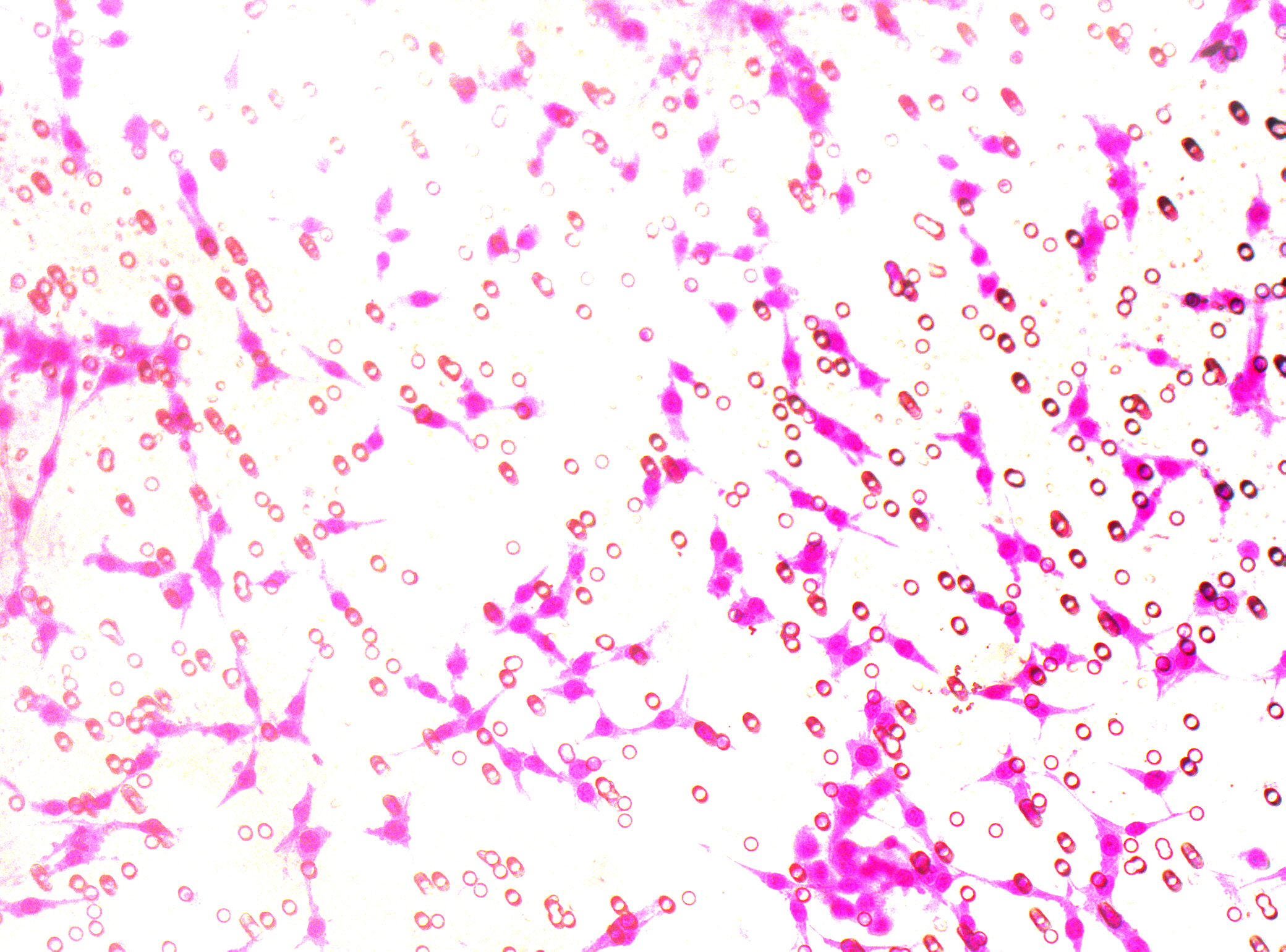

Supplement: S1 File — (ZIP) [file pone.0135508.s001.zip › figure1a/Figure1A-2/8Gy-1.jpg]

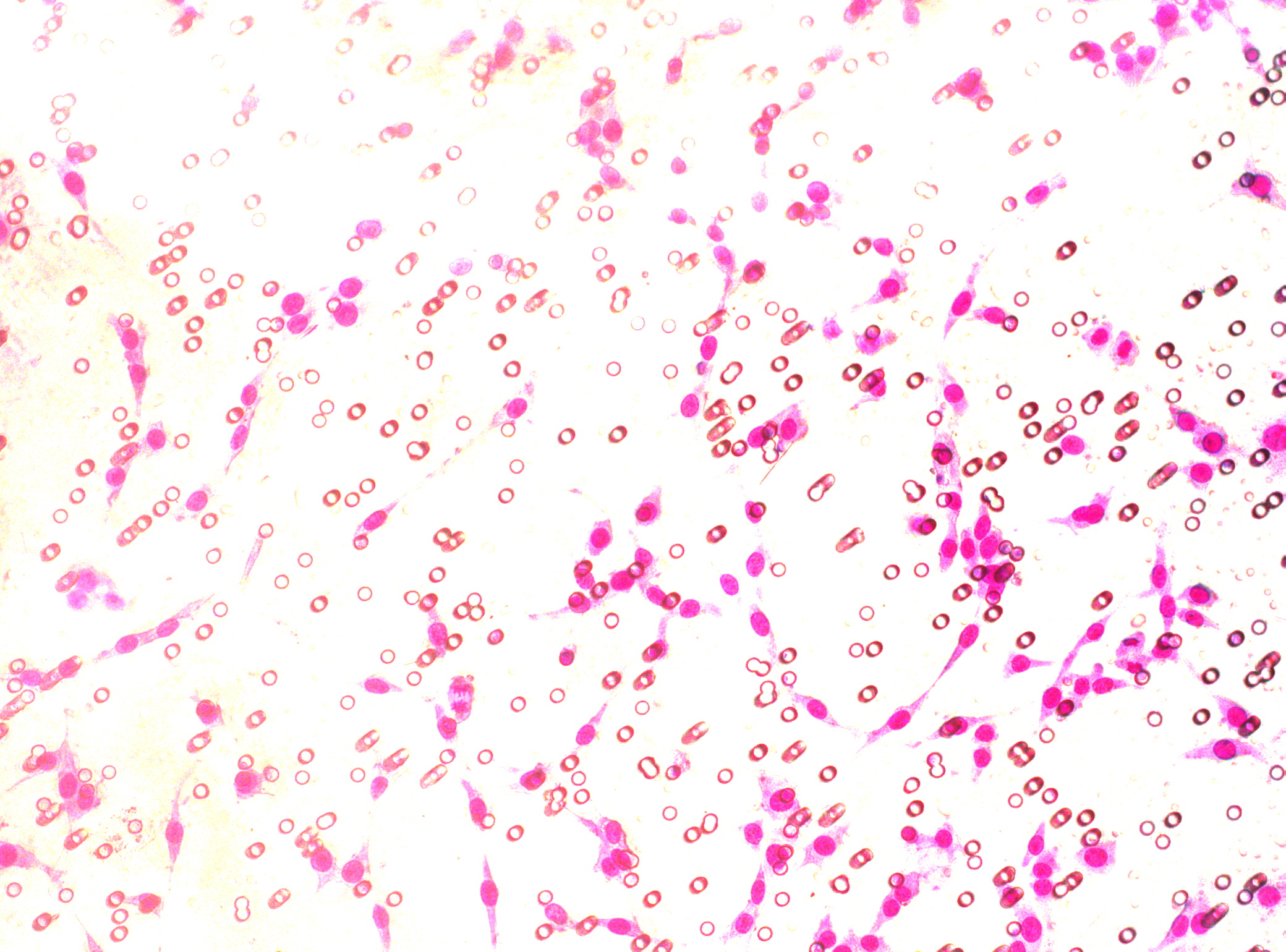

Supplement: S1 File — (ZIP) [file pone.0135508.s001.zip › figure1a/Figure1A-2/2Gy-2.jpg]

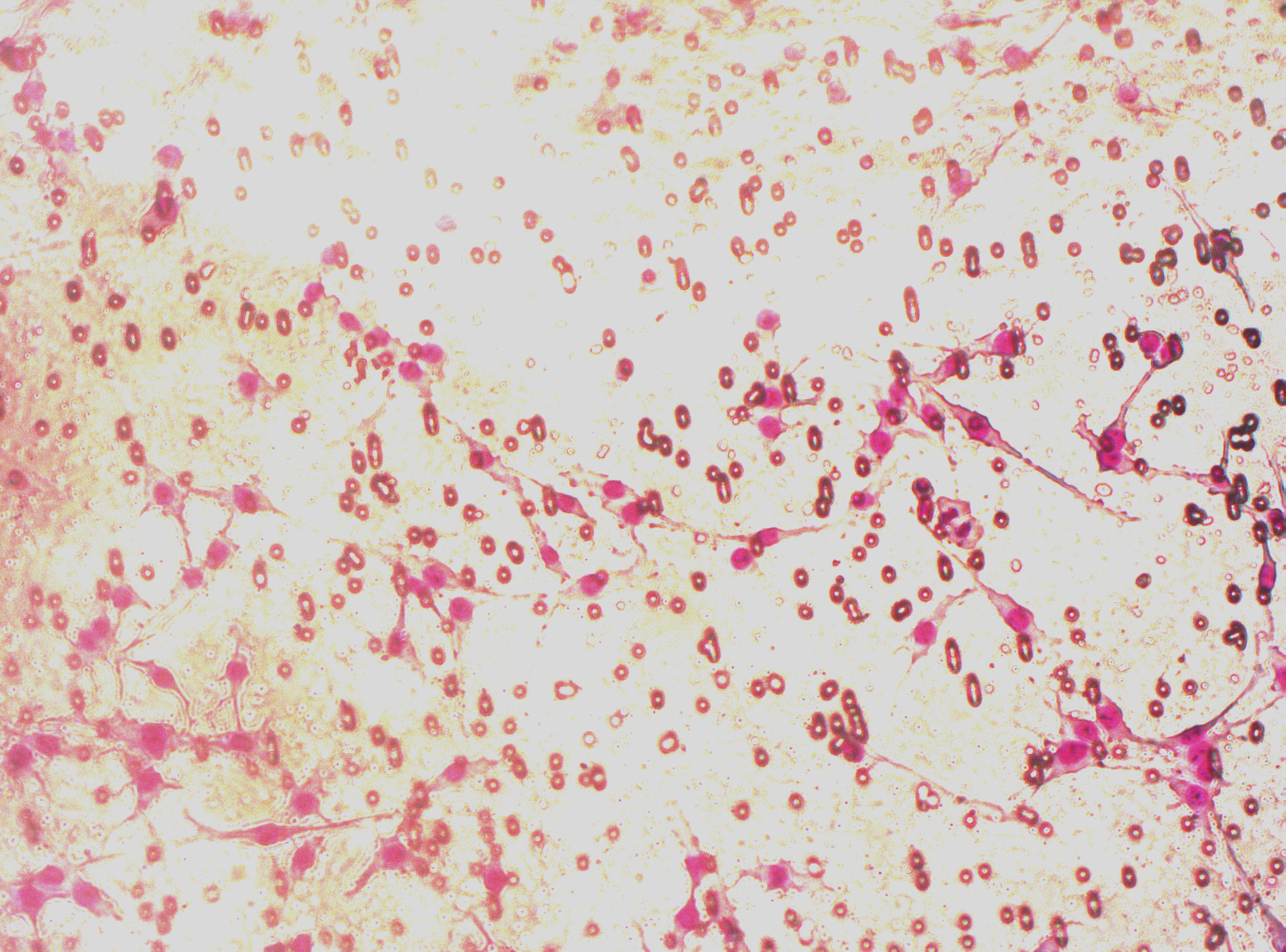

Supplement: S1 File — (ZIP) [file pone.0135508.s001.zip › figure1a/Figure1A-2/4Gy-1.jpg]

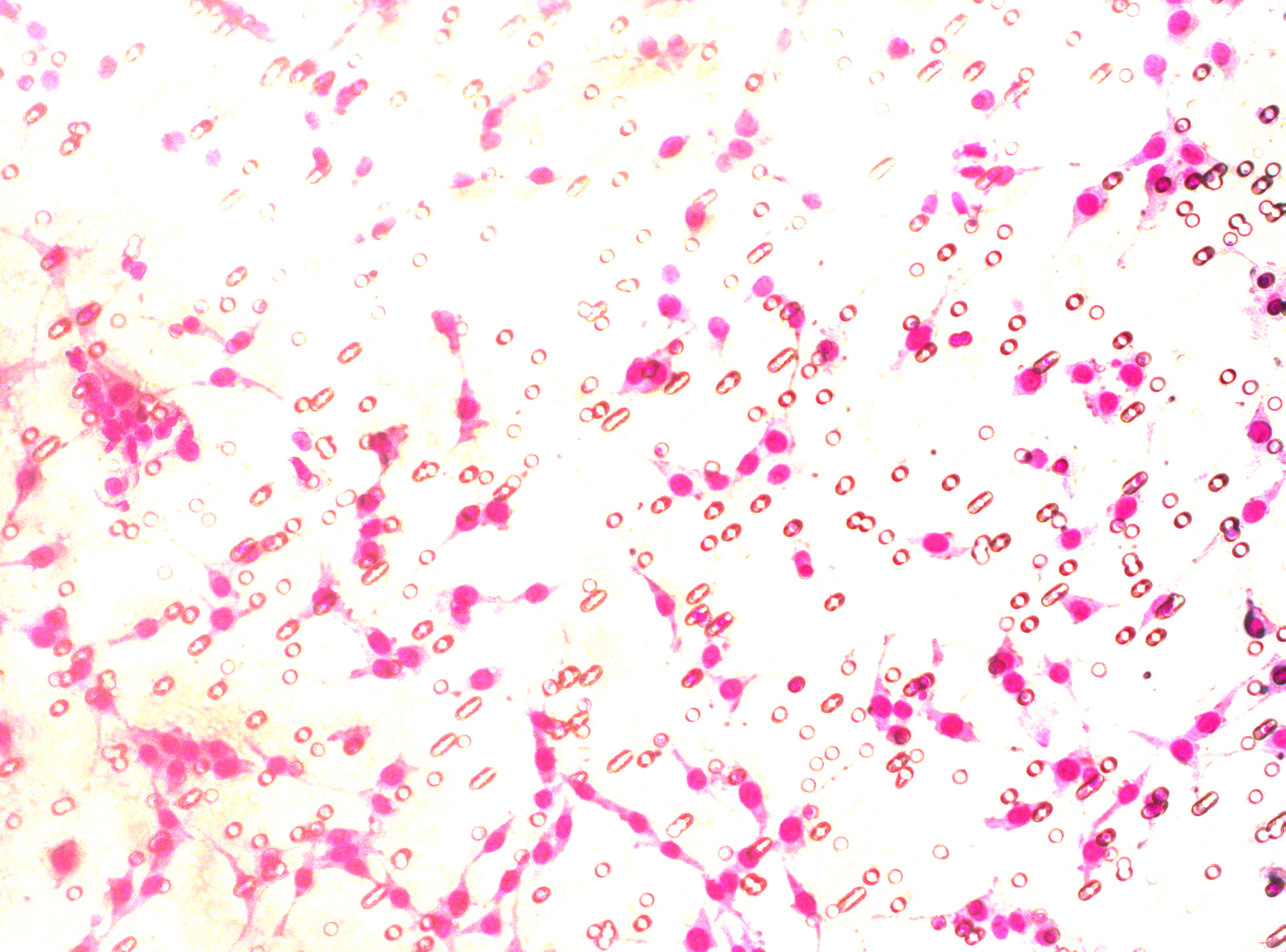

Supplement: S1 File — (ZIP) [file pone.0135508.s001.zip › figure1a/Figure1A-2/2Gy-1.jpg]

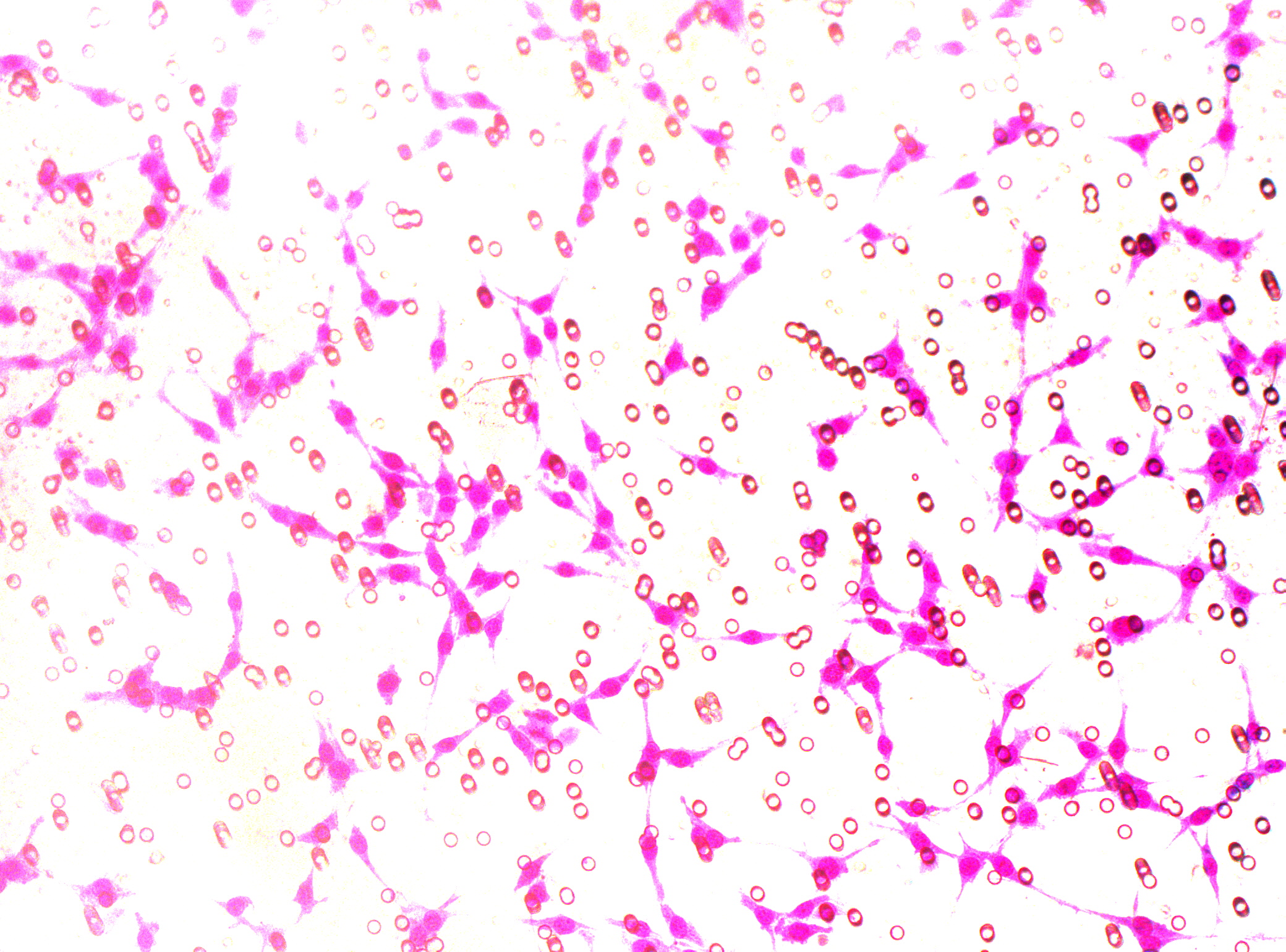

Supplement: S1 File — (ZIP) [file pone.0135508.s001.zip › figure1a/Figure1A-2/0Gy-1.jpg]

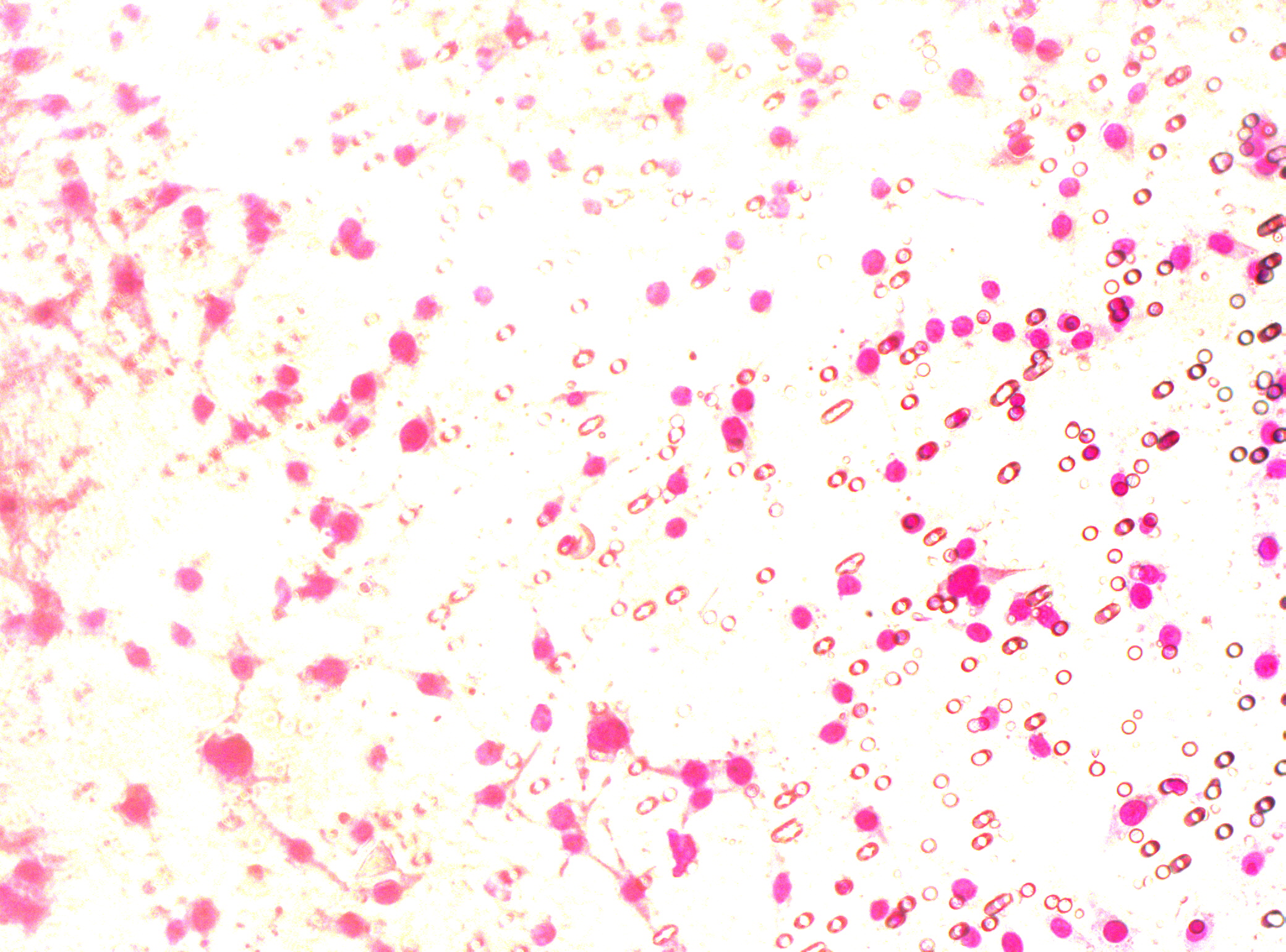

Supplement: S1 File — (ZIP) [file pone.0135508.s001.zip › figure1a/Figure1A-2/4Gy-2.jpg]

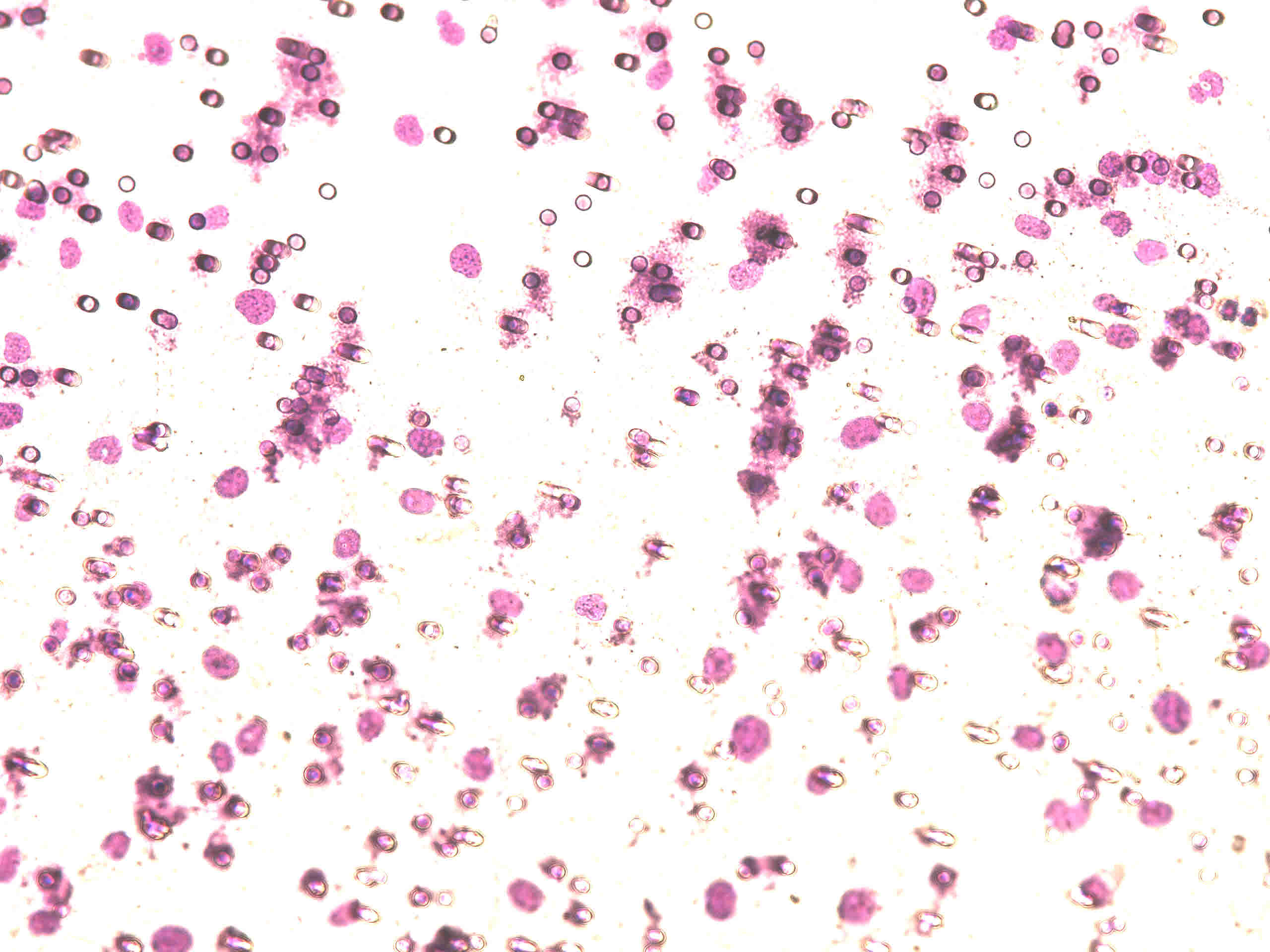

Supplement: S1 File — (ZIP) [file pone.0135508.s001.zip › figure2a/Figure2A-2/8Gy-2.jpg]

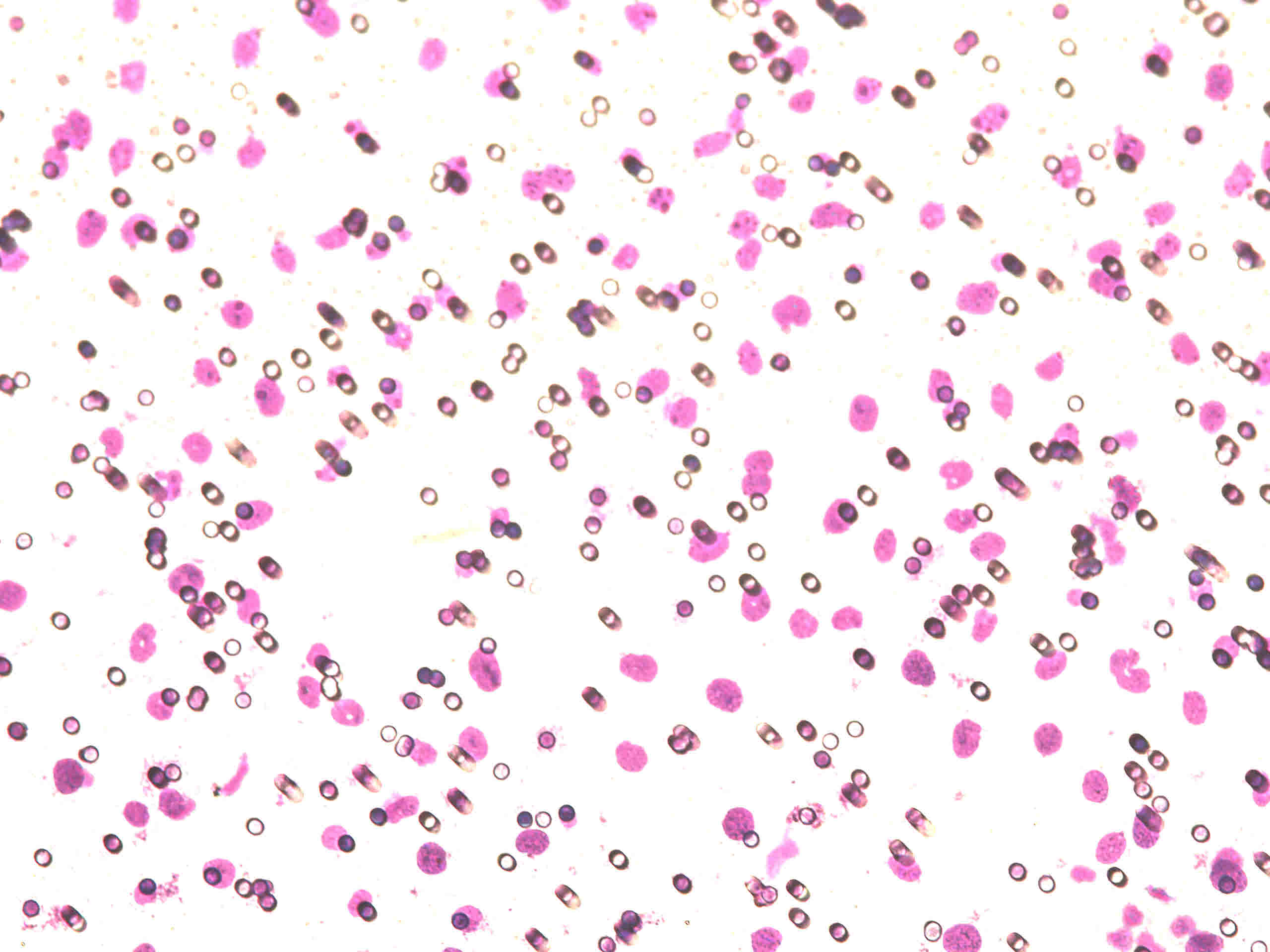

Supplement: S1 File — (ZIP) [file pone.0135508.s001.zip › figure2a/Figure2A-2/8Gy-1.jpg]

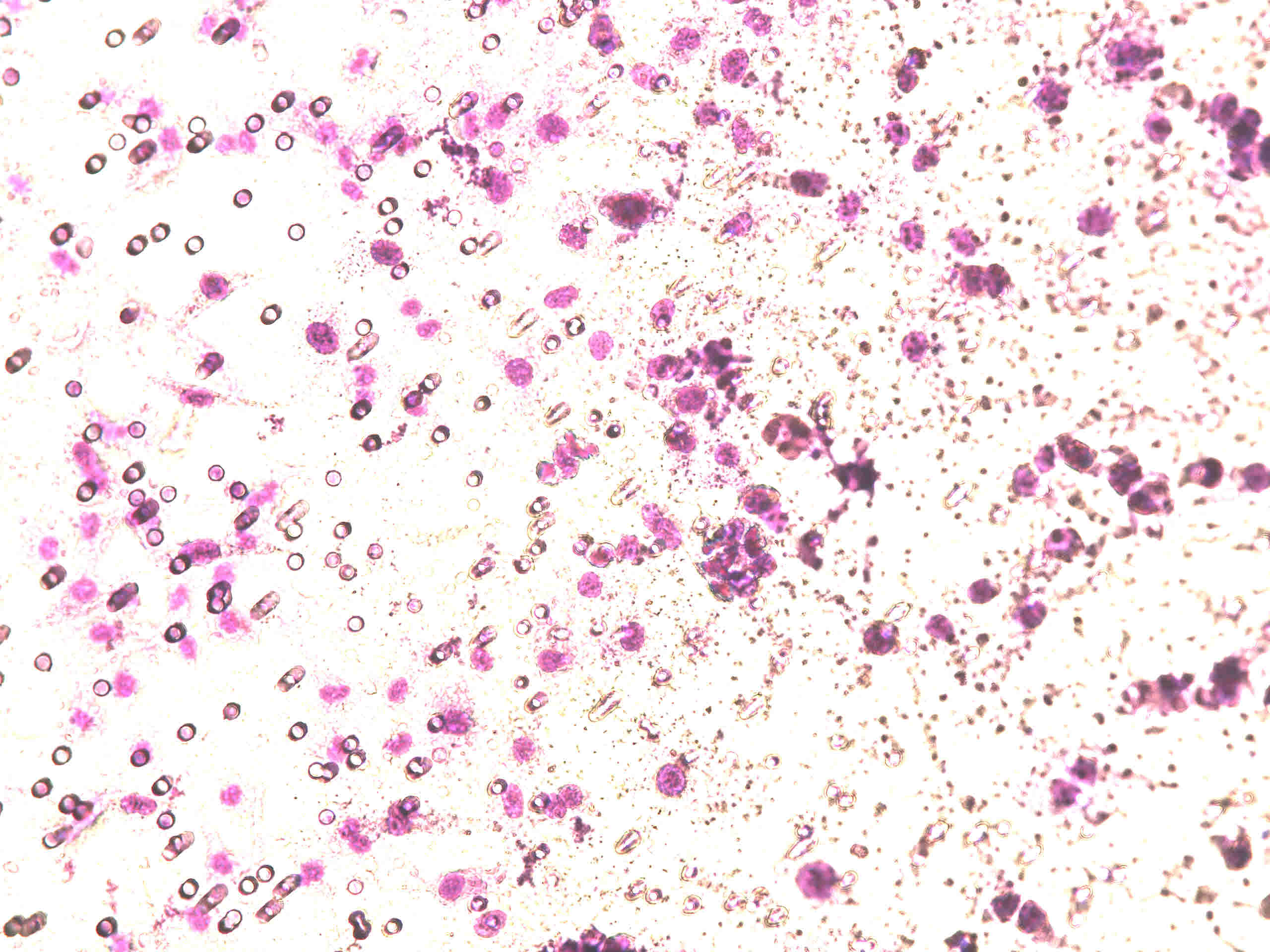

Supplement: S1 File — (ZIP) [file pone.0135508.s001.zip › figure2a/Figure2A-2/2Gy-2.jpg]

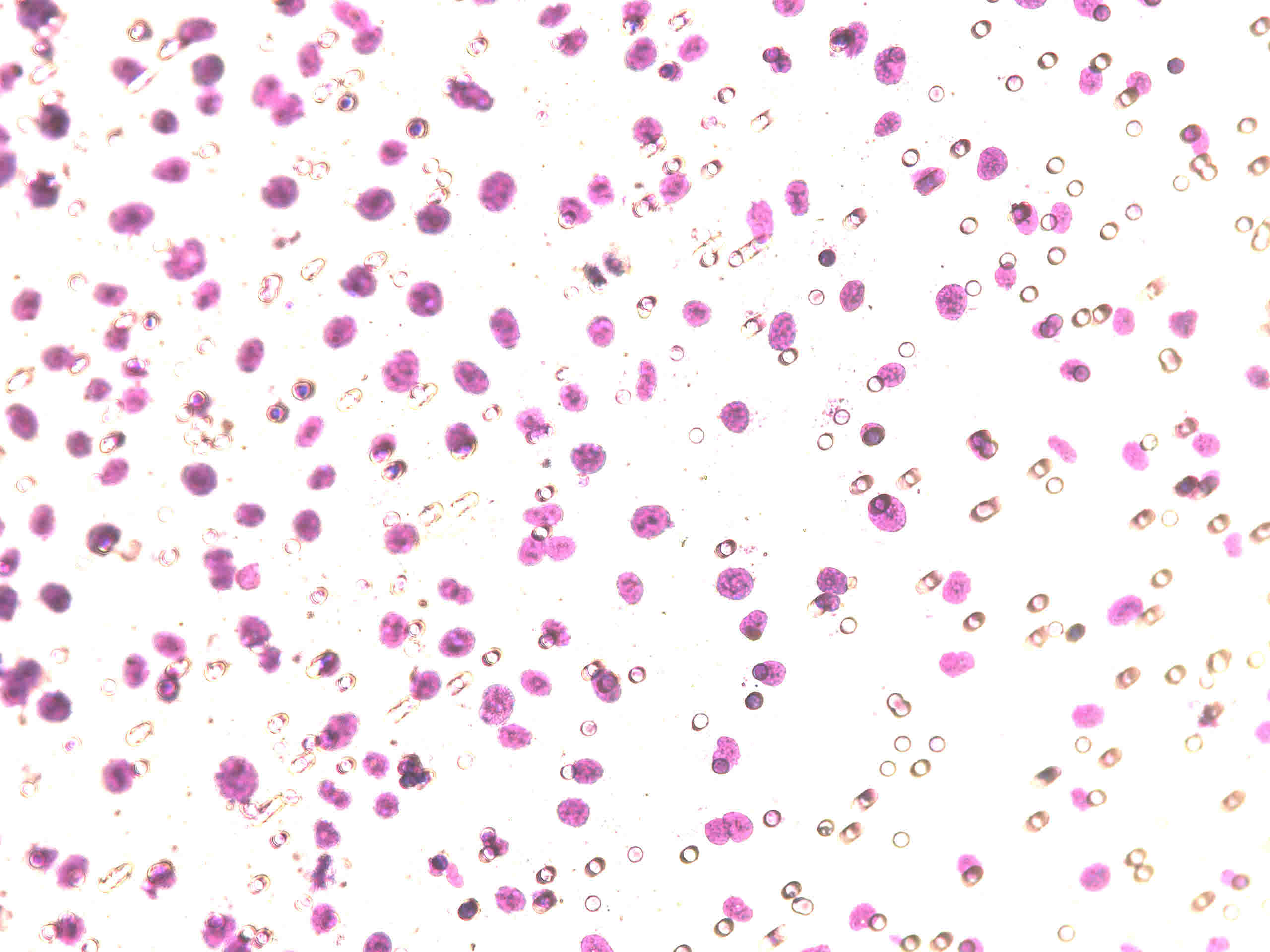

Supplement: S1 File — (ZIP) [file pone.0135508.s001.zip › figure2a/Figure2A-2/0Gy-2.jpg]

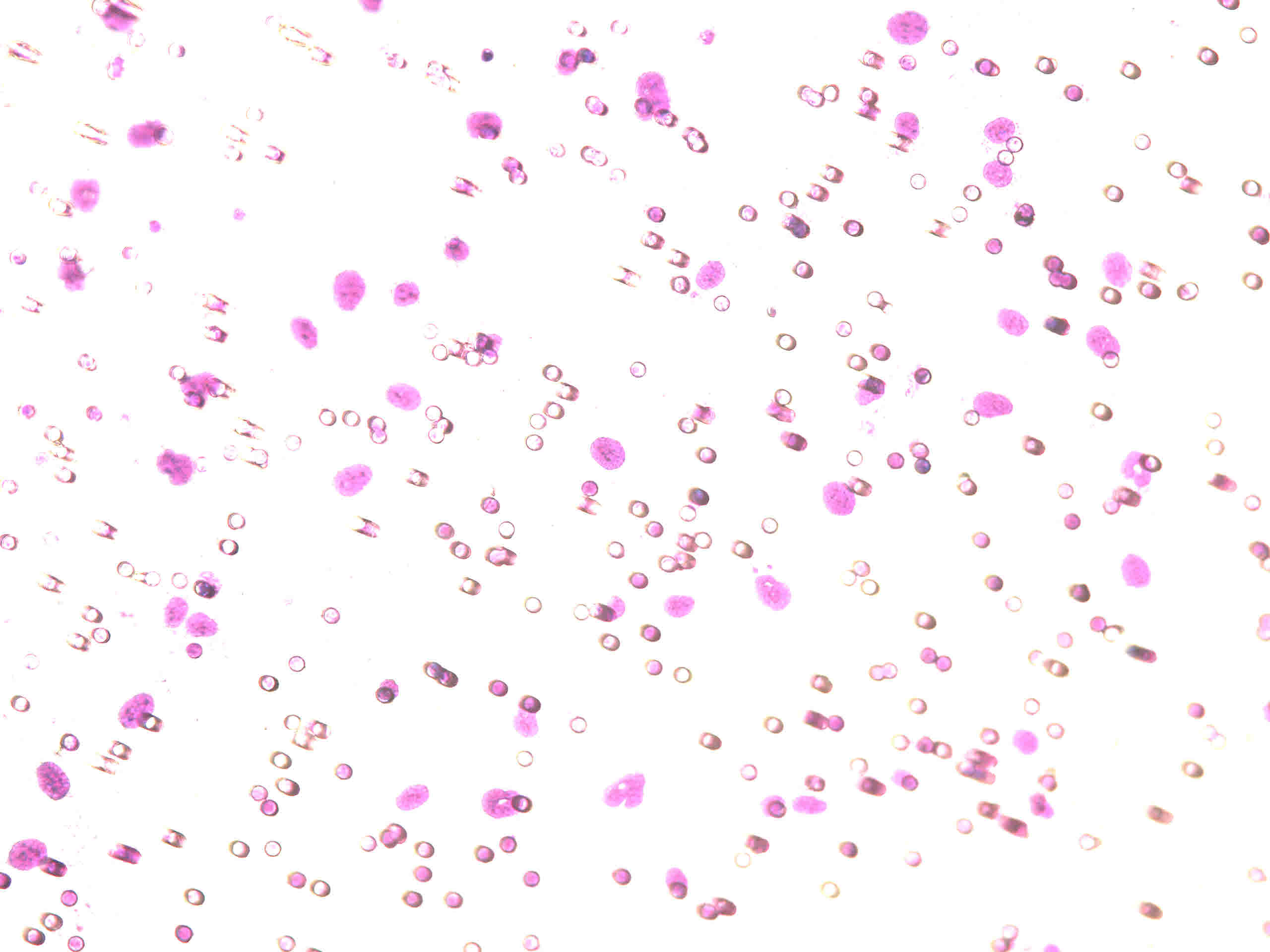

Supplement: S1 File — (ZIP) [file pone.0135508.s001.zip › figure2a/Figure2A-2/4Gy-1.jpg]

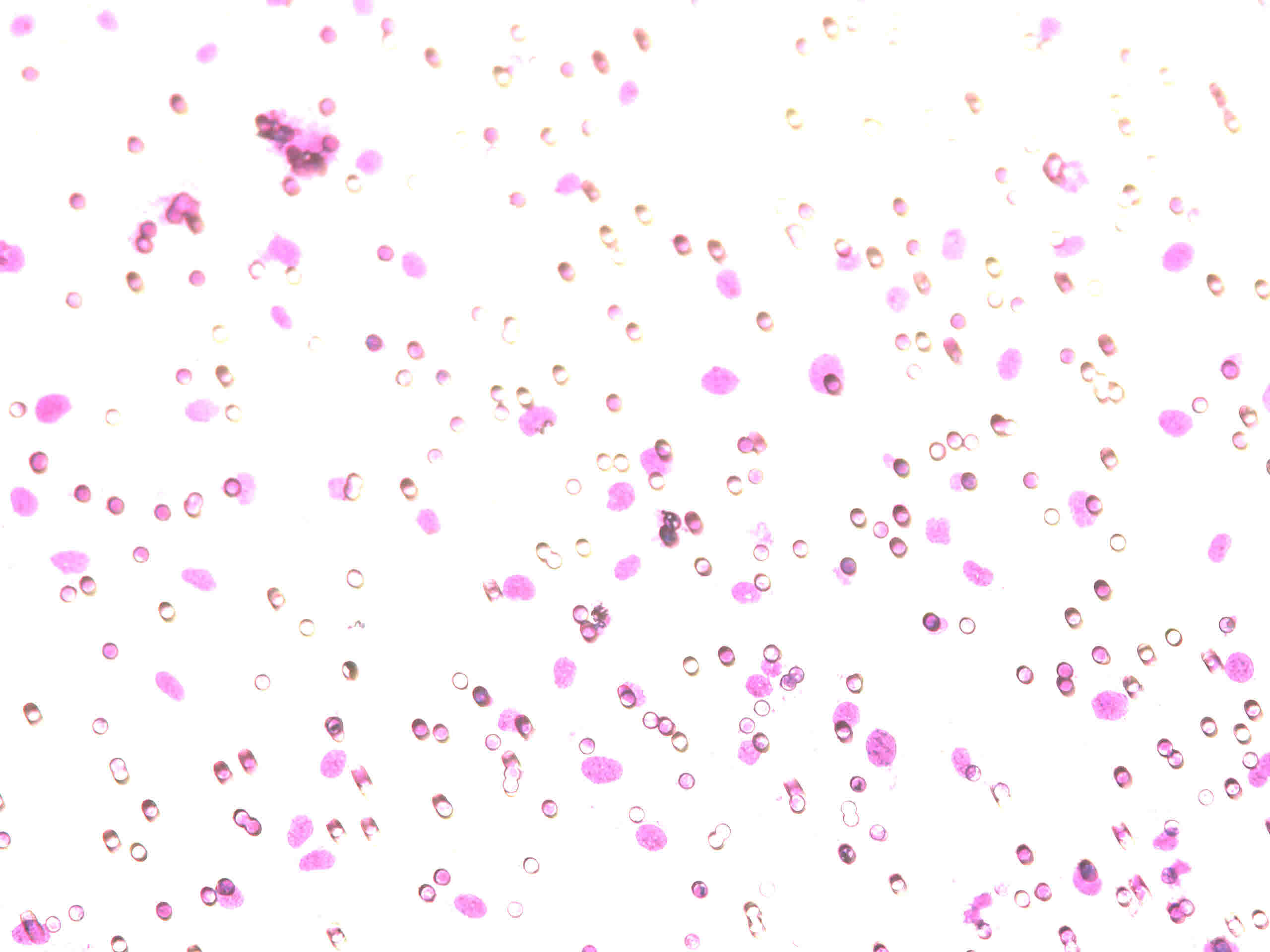

Supplement: S1 File — (ZIP) [file pone.0135508.s001.zip › figure2a/Figure2A-2/2Gy-1.jpg]

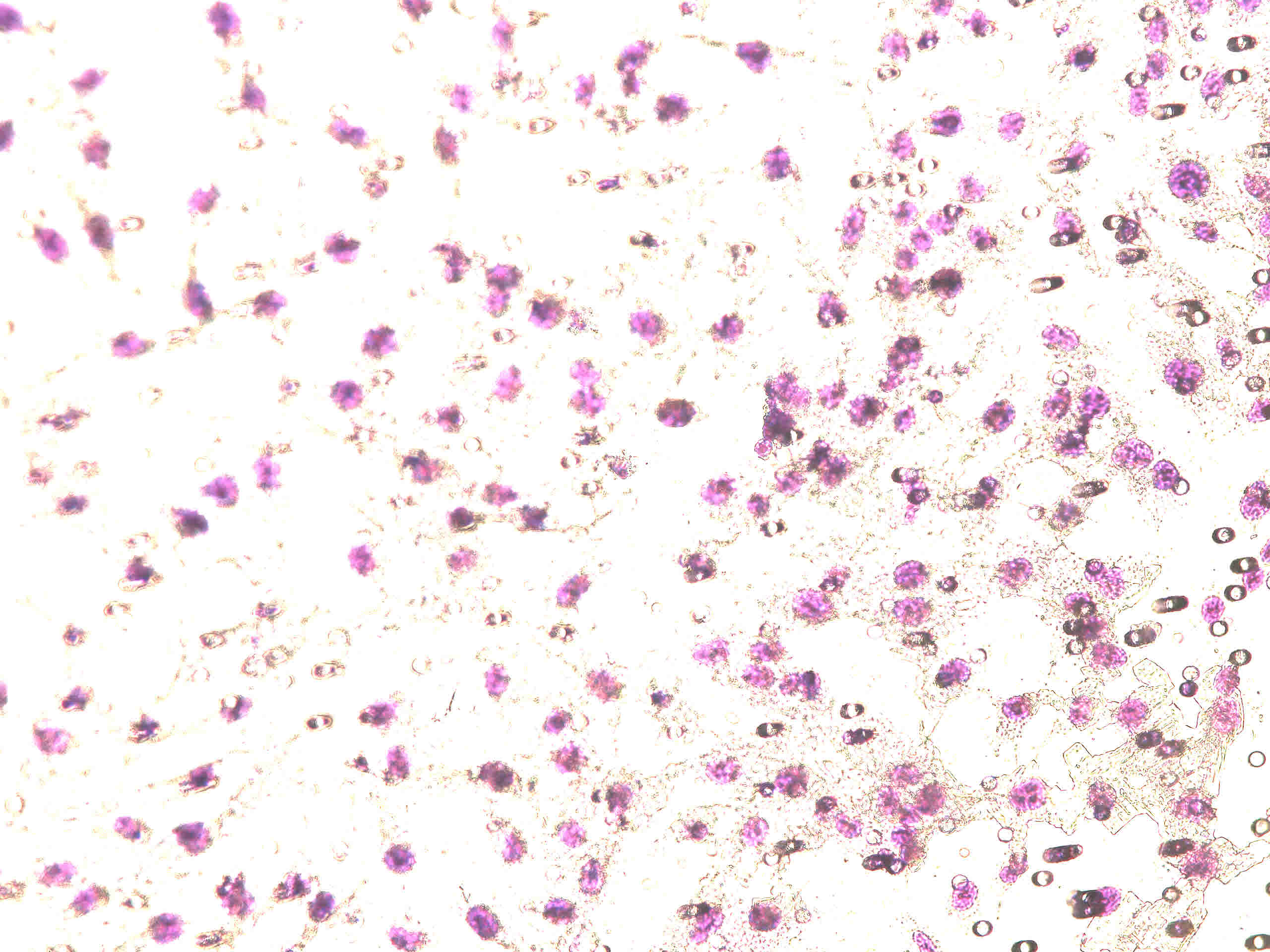

Supplement: S1 File — (ZIP) [file pone.0135508.s001.zip › figure2a/Figure2A-2/0Gy-1.jpg]

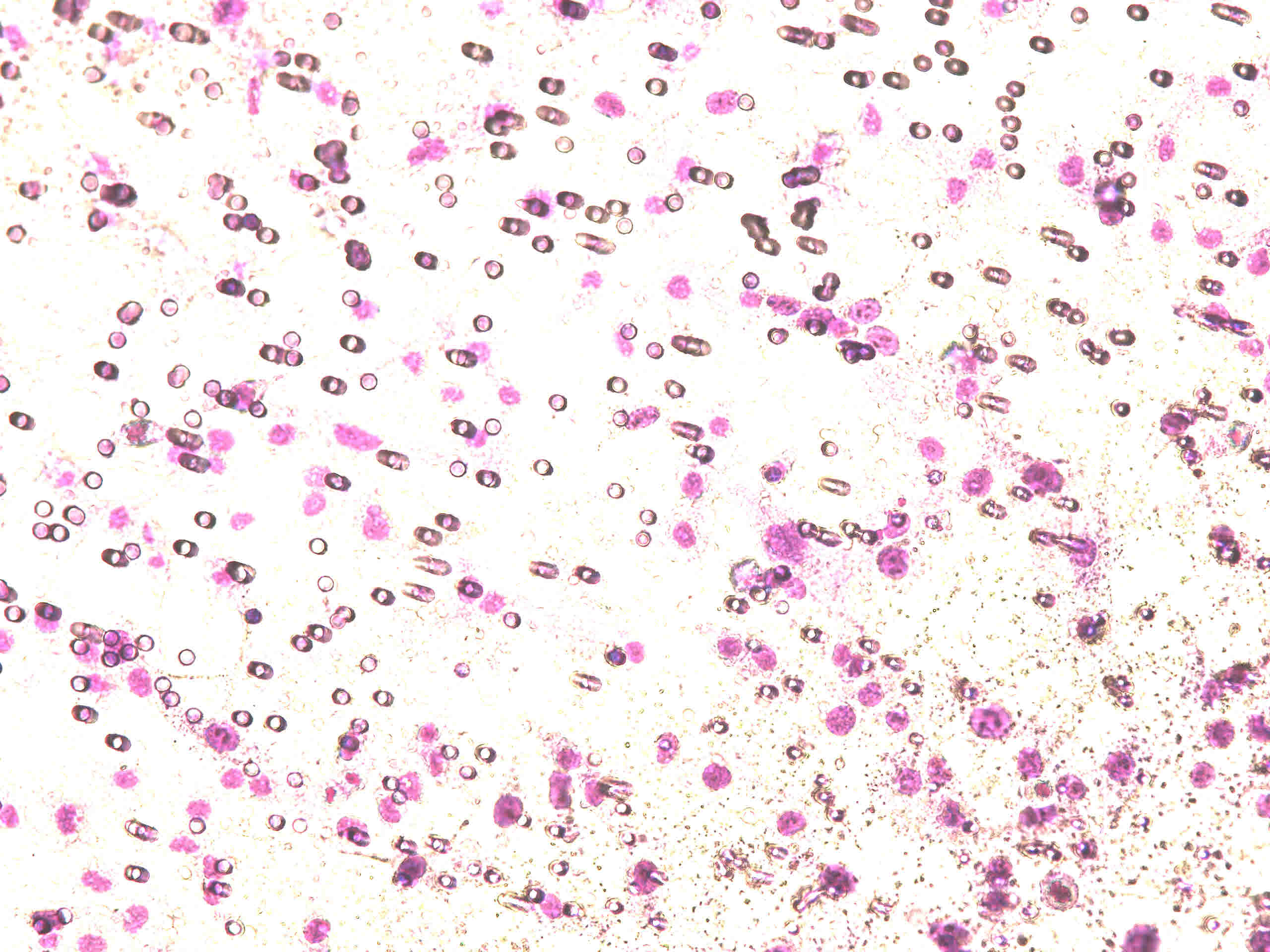

Supplement: S1 File — (ZIP) [file pone.0135508.s001.zip › figure2a/Figure2A-2/4Gy-2.jpg]

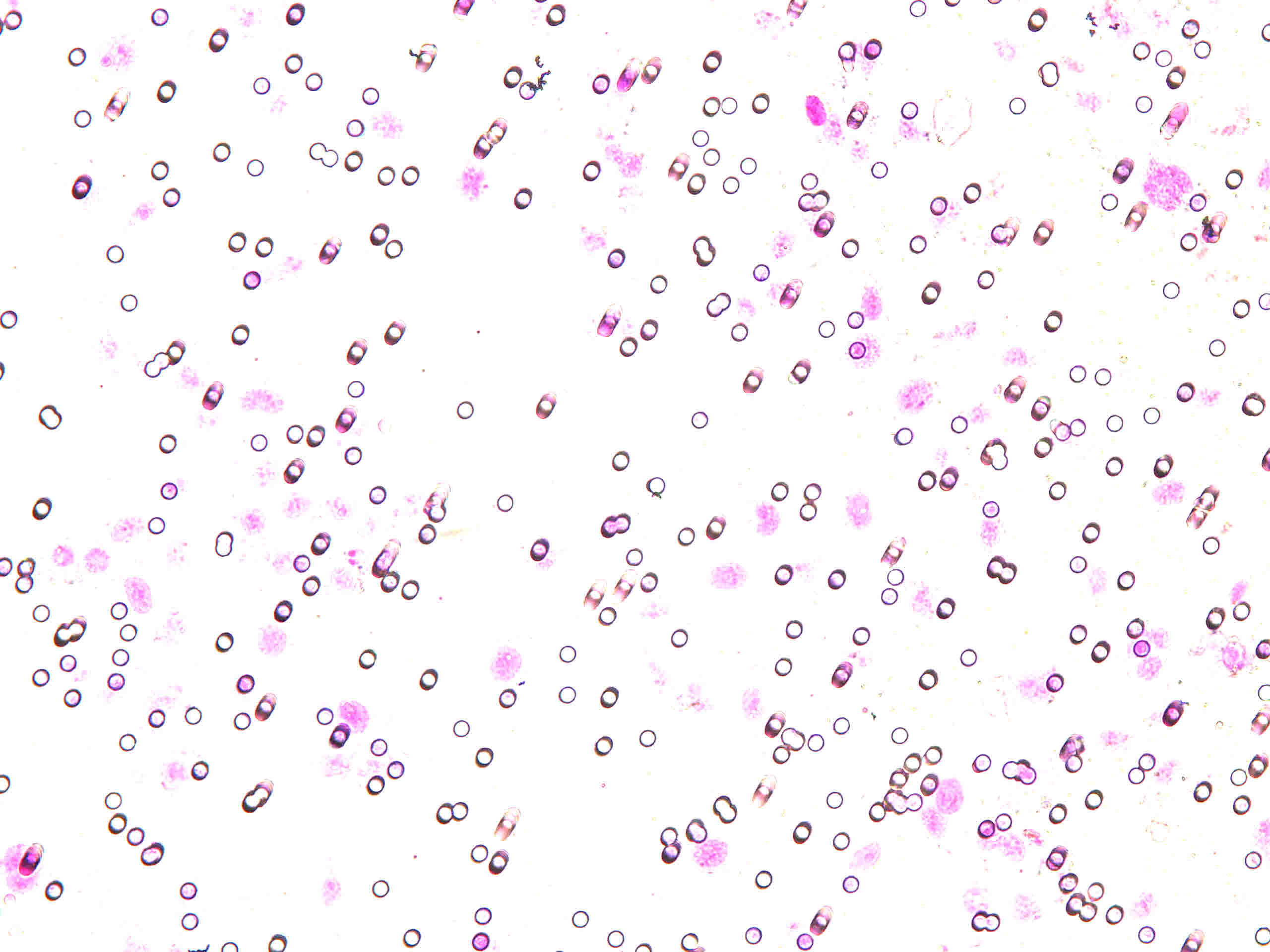

Supplement: S1 File — (ZIP) [file pone.0135508.s001.zip › figure2a/Figure2A-3/2Gy-1.jpg]

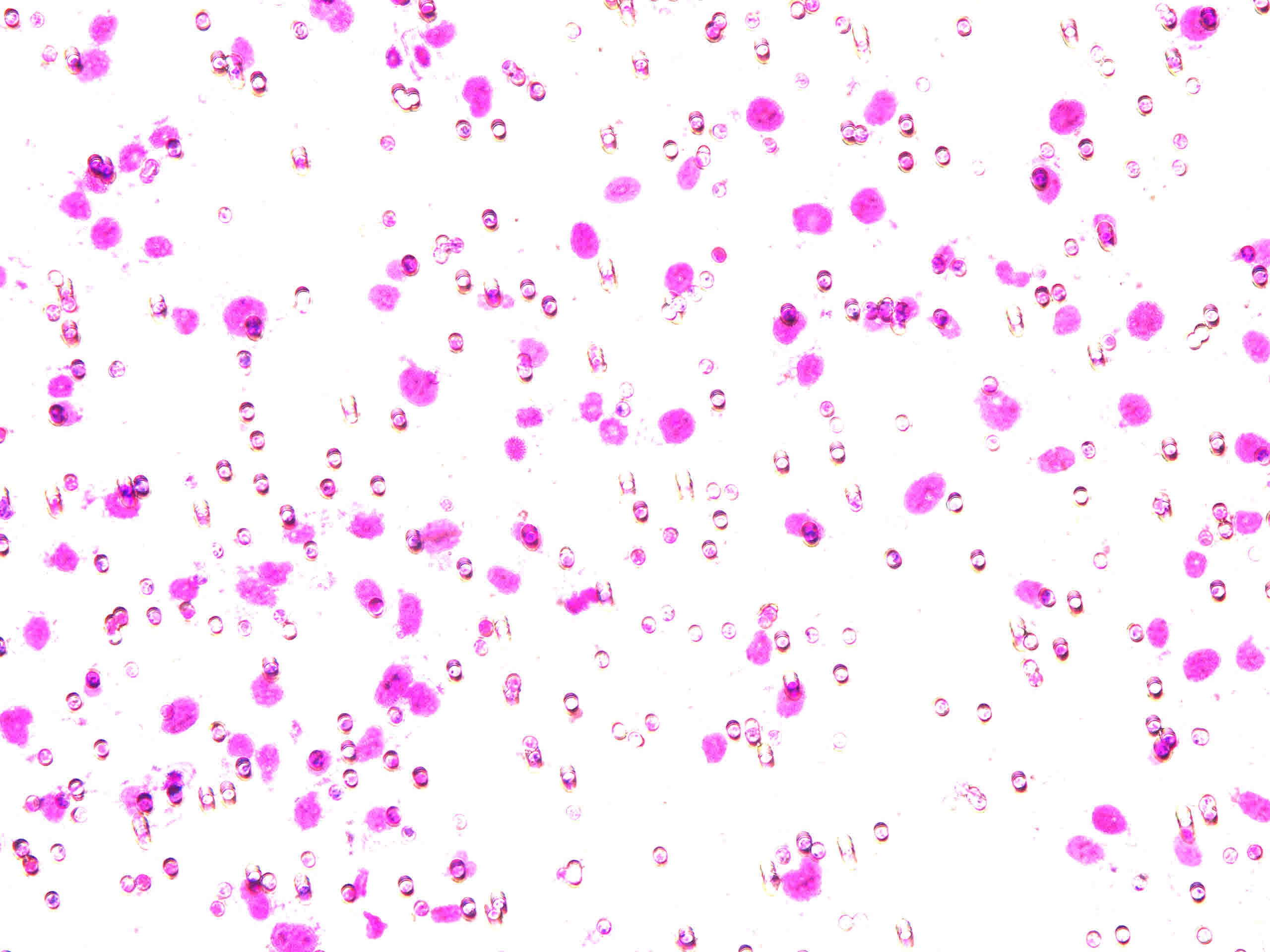

Supplement: S1 File — (ZIP) [file pone.0135508.s001.zip › figure2a/Figure2A-3/0Gy-1.jpg]

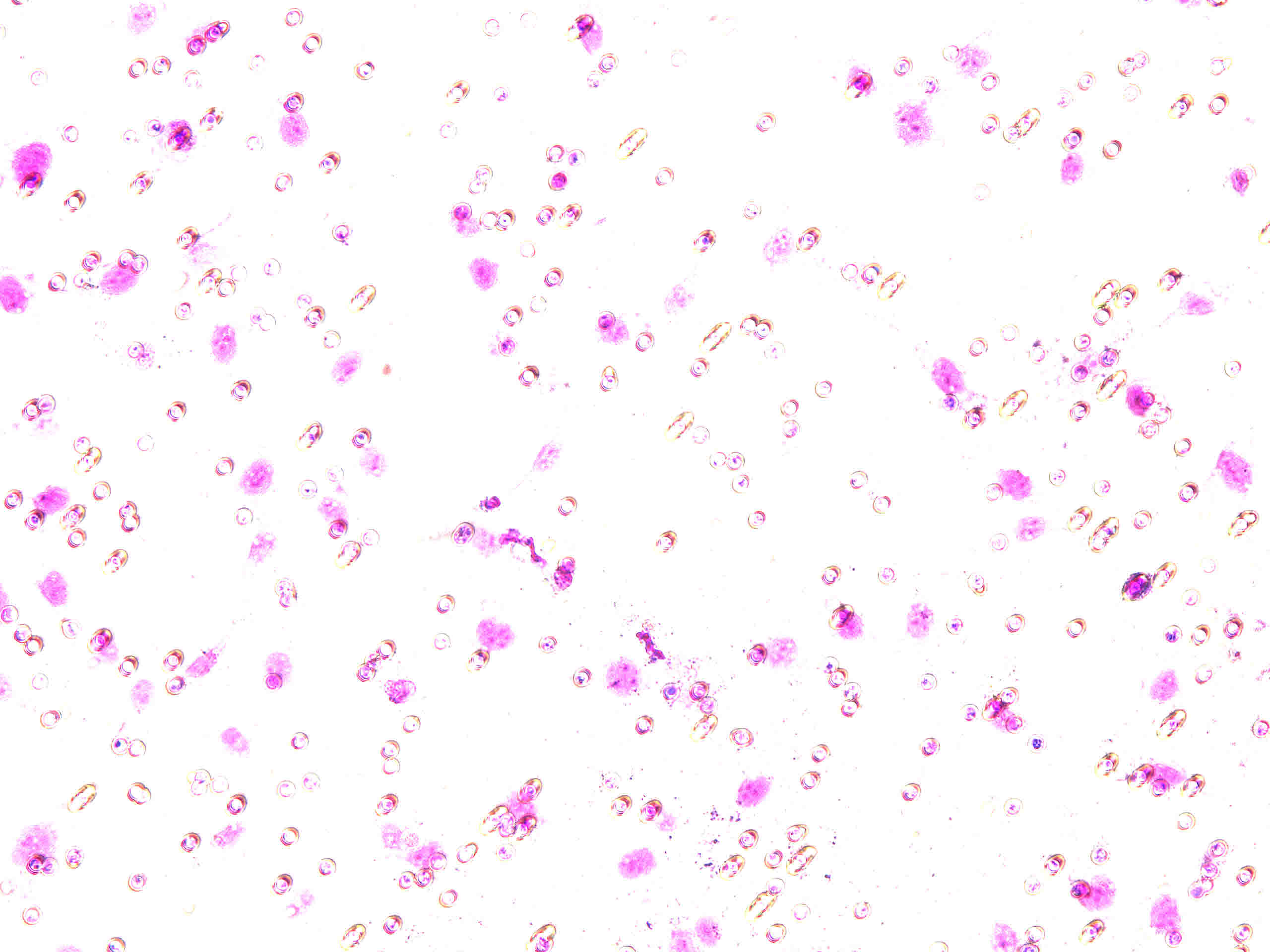

Supplement: S1 File — (ZIP) [file pone.0135508.s001.zip › figure2a/Figure2A-3/4Gy-2.jpg]

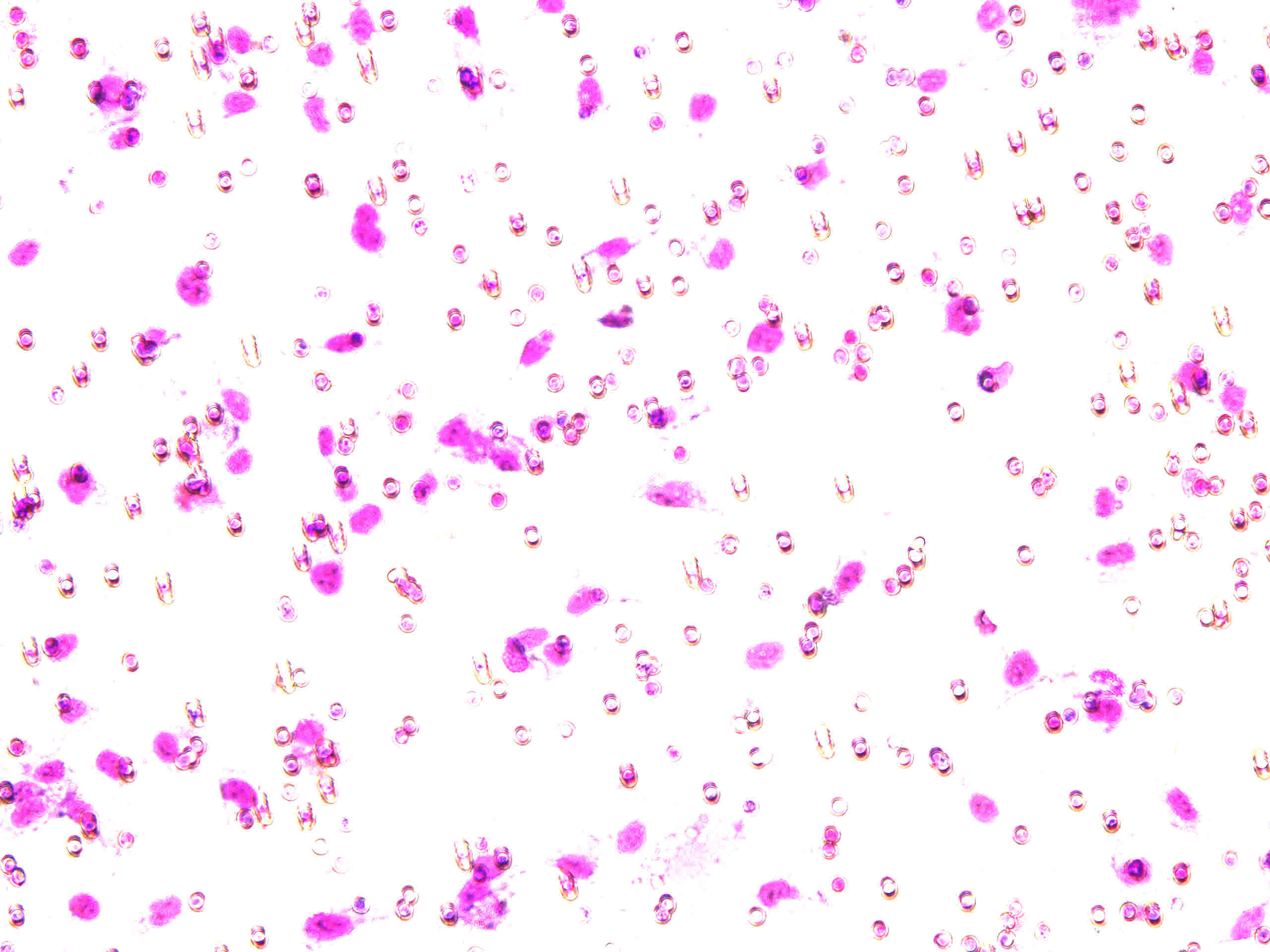

Supplement: S1 File — (ZIP) [file pone.0135508.s001.zip › figure2a/Figure2A-3/0Gy-2.jpg]

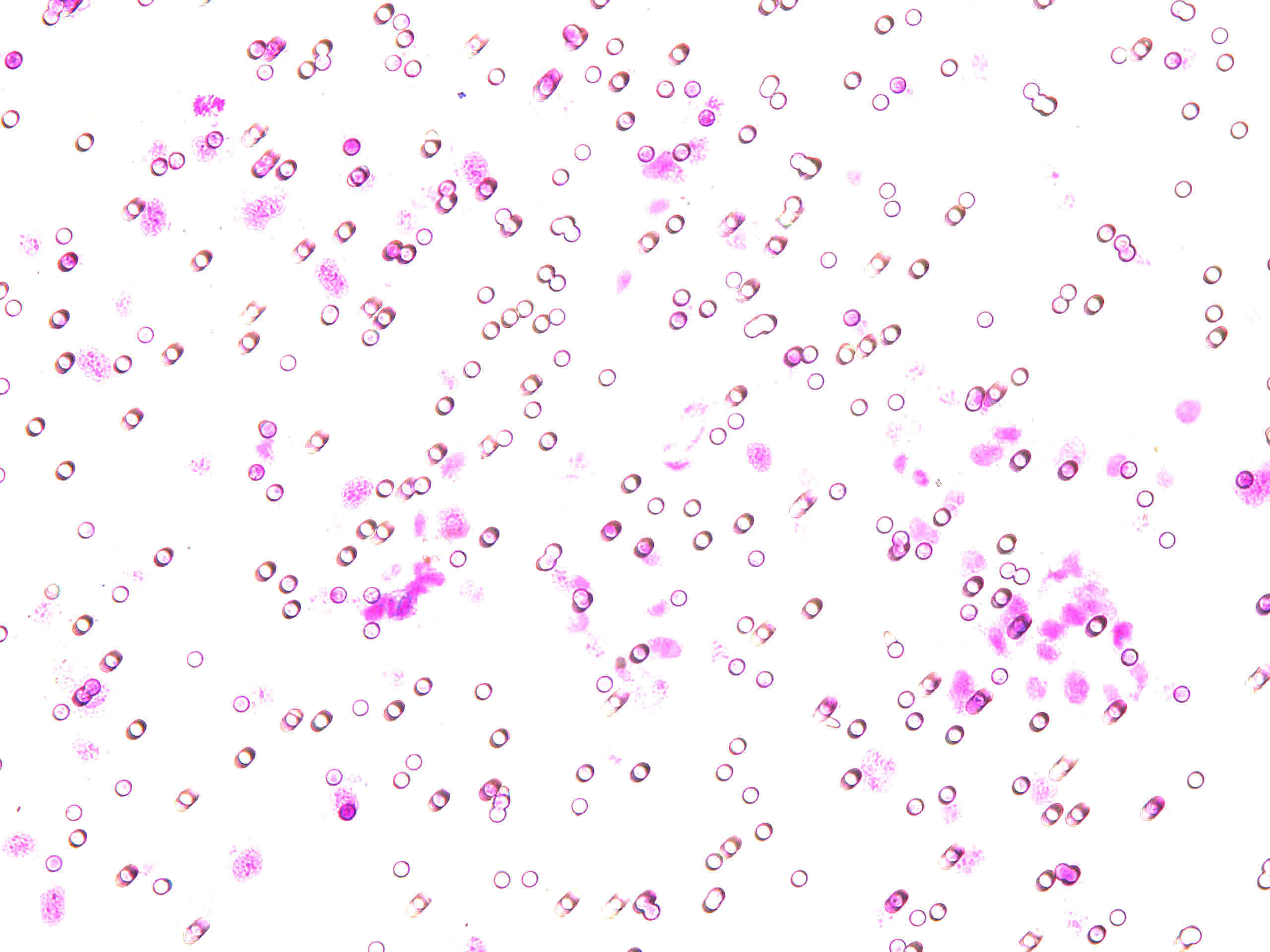

Supplement: S1 File — (ZIP) [file pone.0135508.s001.zip › figure2a/Figure2A-3/4Gy-1.jpg]

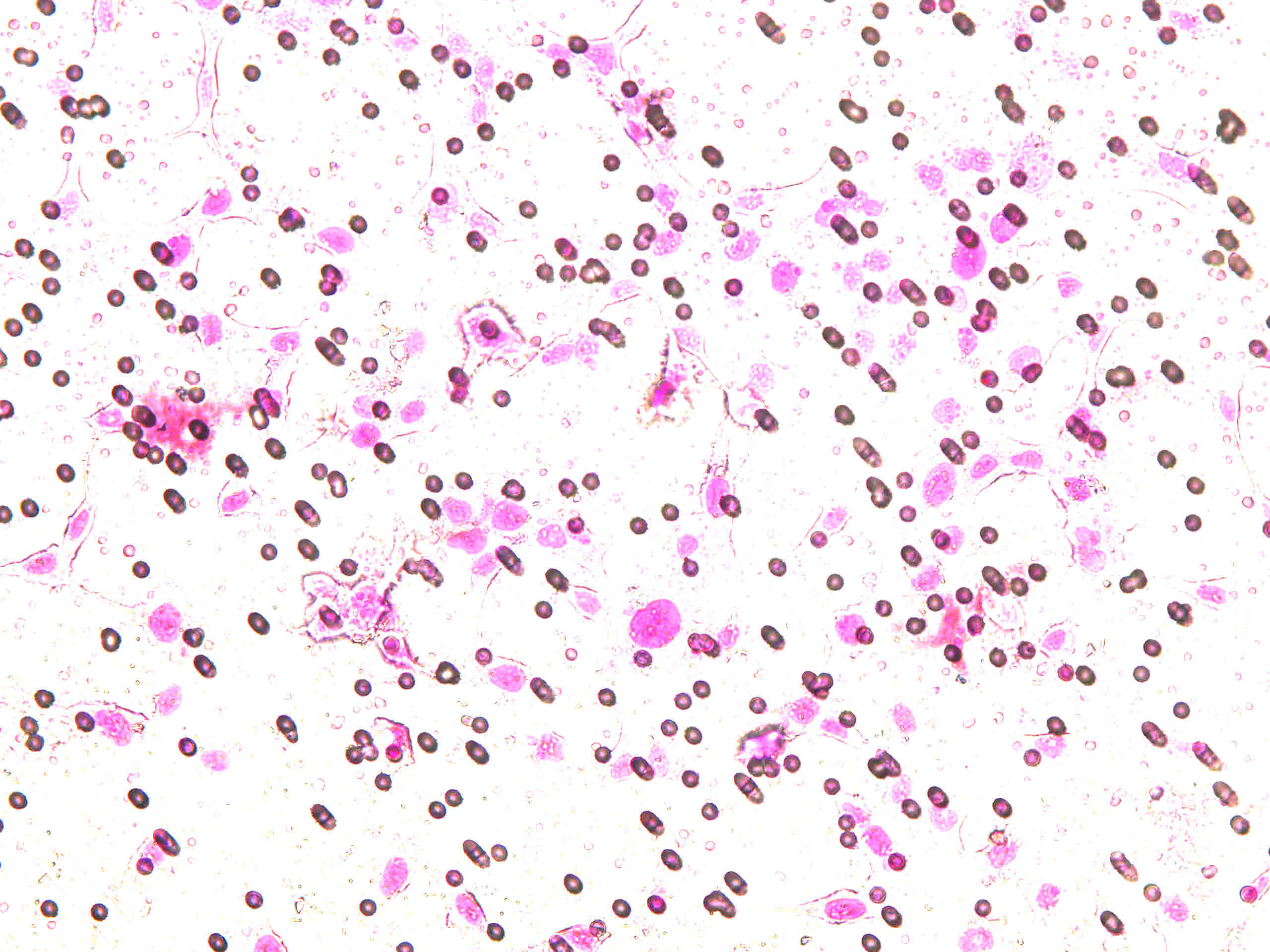

Supplement: S1 File — (ZIP) [file pone.0135508.s001.zip › figure2a/Figure2A-3/8Gy-1.jpg]

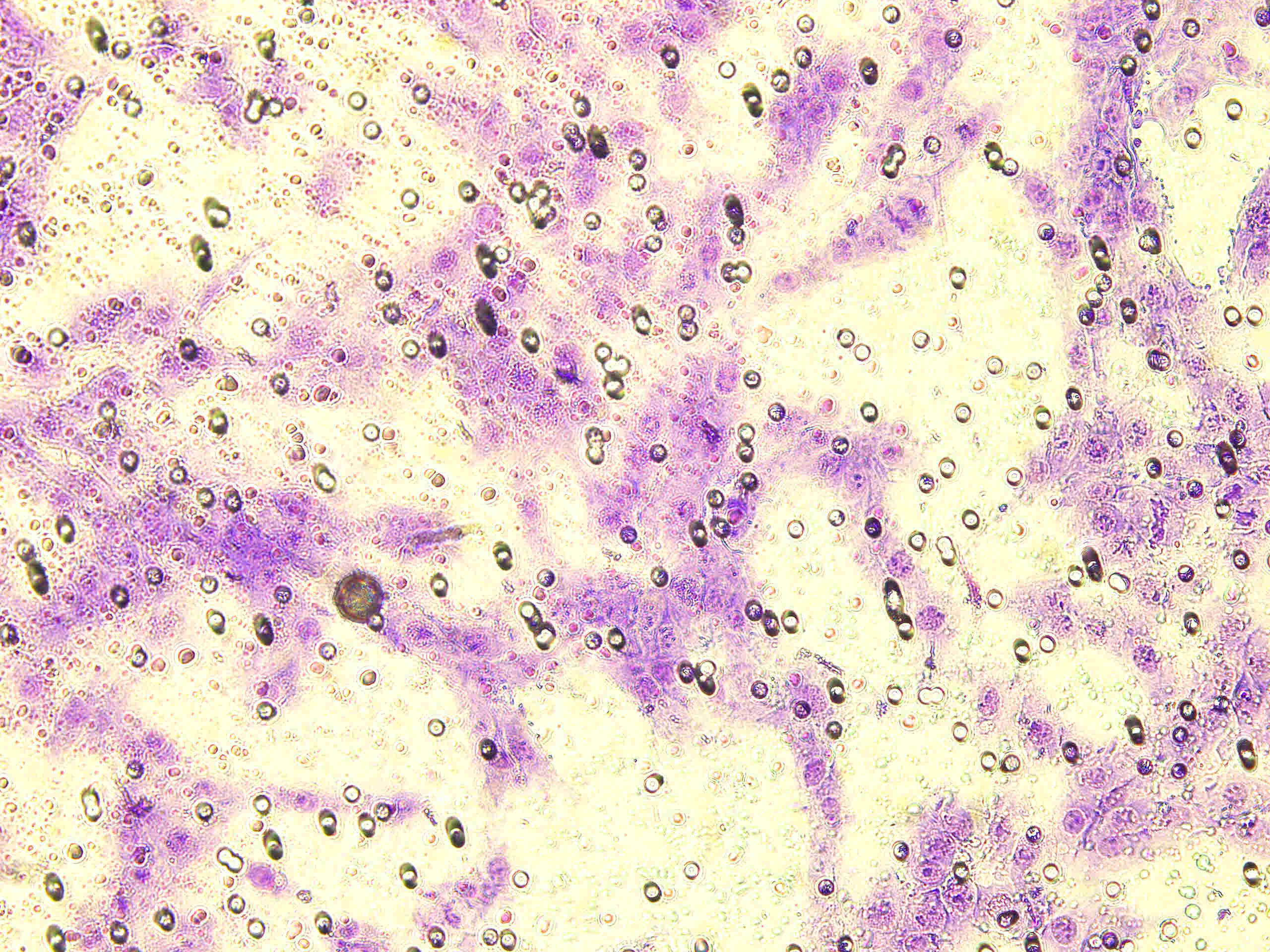

Supplement: S1 File — (ZIP) [file pone.0135508.s001.zip › figure1a/Figure1A-1/2Gy-2.jpg]

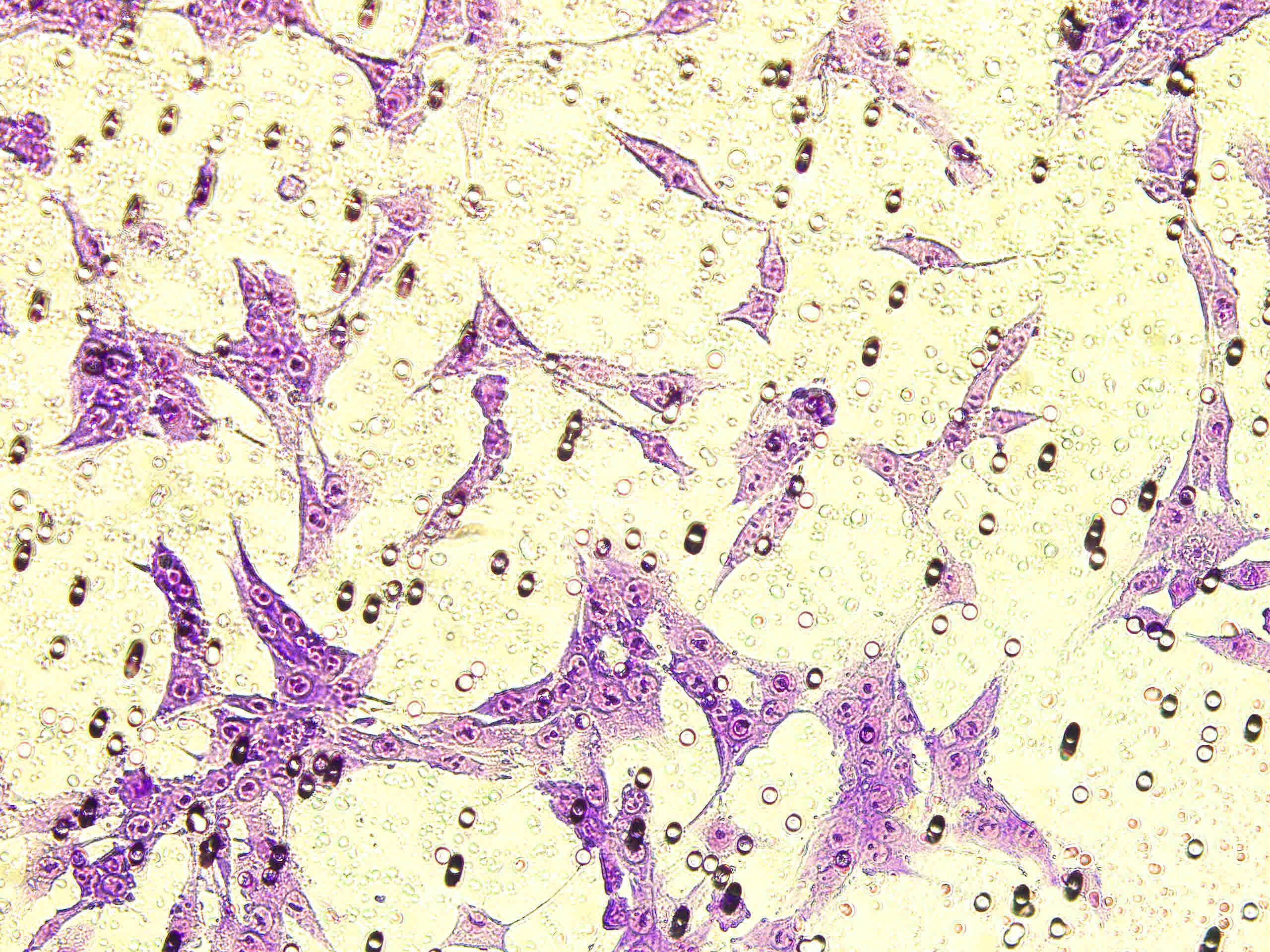

Supplement: S1 File — (ZIP) [file pone.0135508.s001.zip › figure1a/Figure1A-1/4Gy-1.jpg]

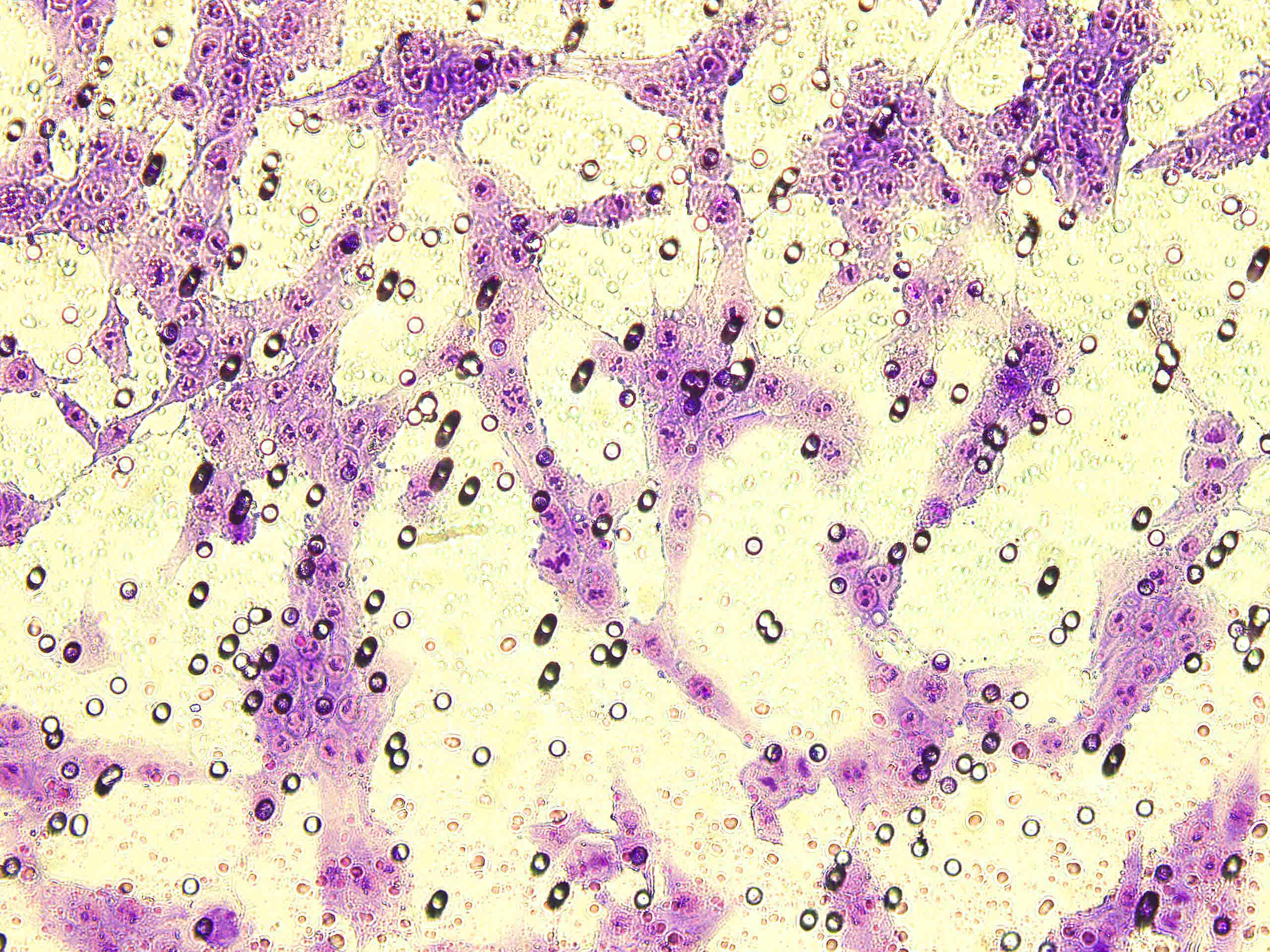

Supplement: S1 File — (ZIP) [file pone.0135508.s001.zip › figure1a/Figure1A-1/2Gy-1.jpg]

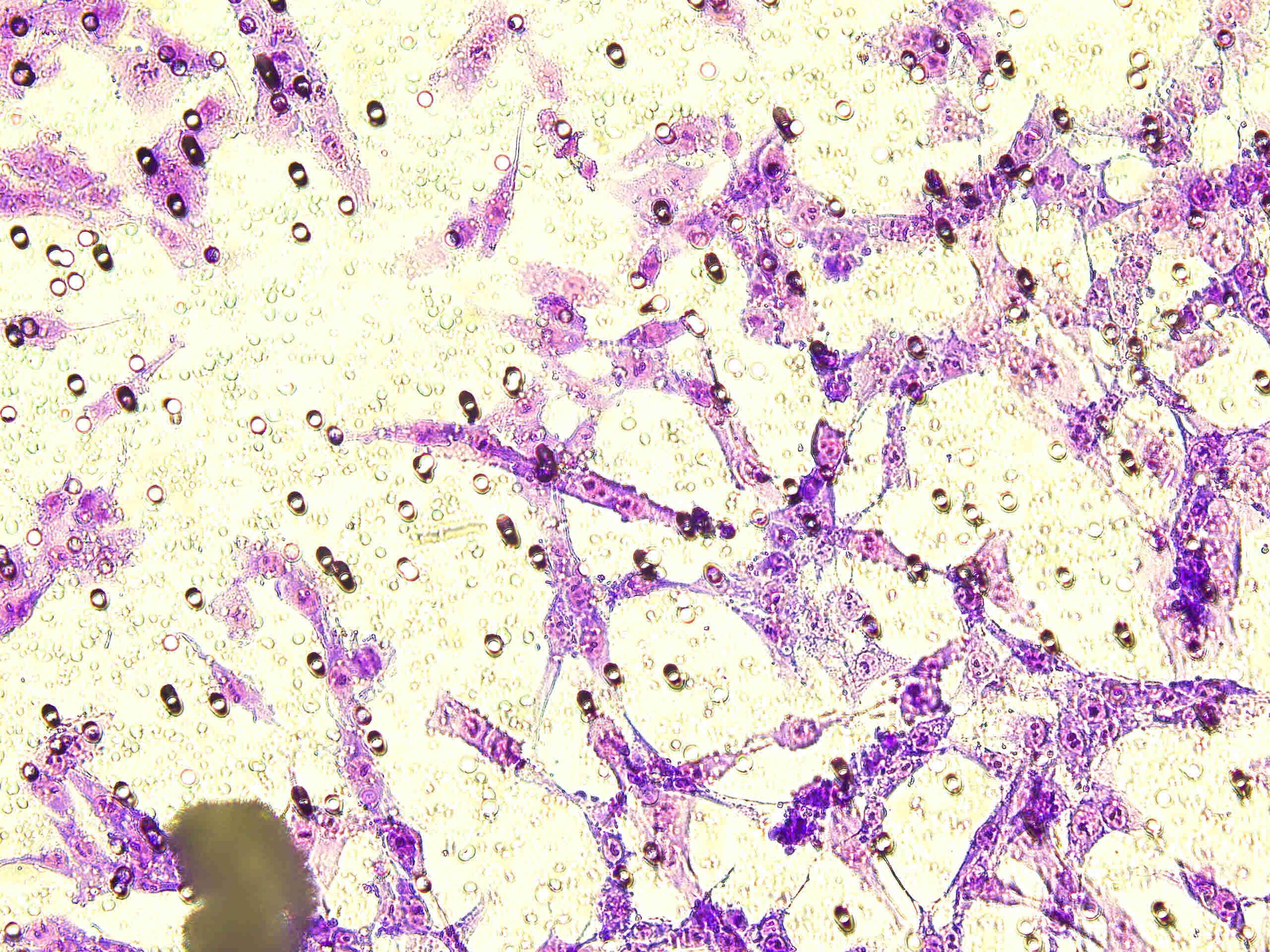

Supplement: S1 File — (ZIP) [file pone.0135508.s001.zip › figure1a/Figure1A-1/0Gy-1.jpg]

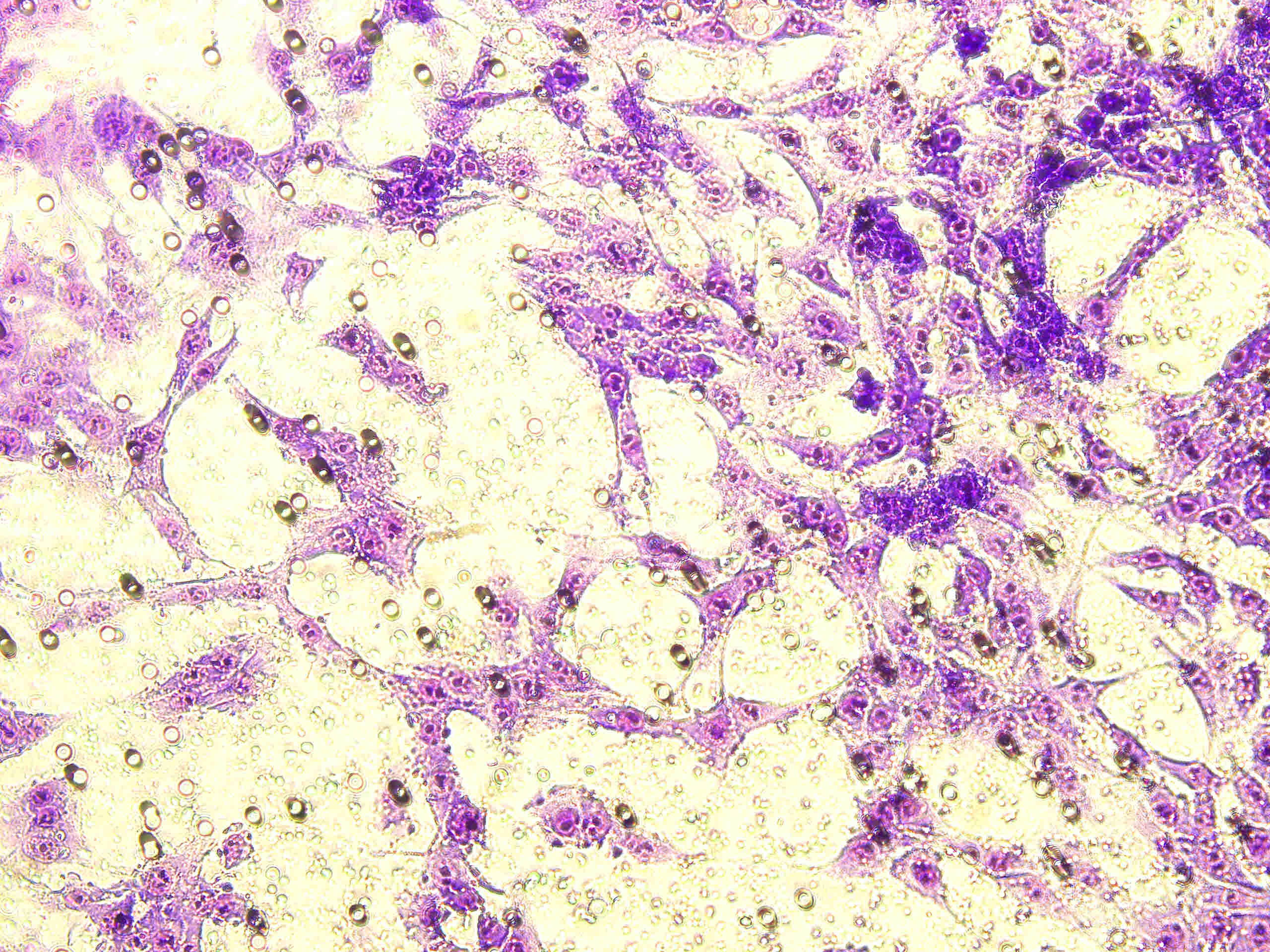

Supplement: S1 File — (ZIP) [file pone.0135508.s001.zip › figure1a/Figure1A-1/8Gy-1.jpg]
